# The comparative effectiveness and safety of fluticasone-salmeterol via metered-dose versus dry powder inhalers for COPD: A new user cohort study

**DOI:** 10.1371/journal.pmed.1004596

**Published:** 2025-05-14

**Authors:** Brandon J. Demkowicz, Kevin Rader, Shirley V. Wang, Aaron S. Kesselheim, William B. Feldman

**Affiliations:** 1 Department of Statistics, Harvard University, Cambridge, Massachusetts, United States of America; 2 Program on Regulation, Therapeutics, and Law, Division of Pharmacoepidemiology and Pharmacoeconomics, Department of Medicine, Brigham and Women’s Hospital, Boston, Massachusetts, United States of America; 3 Harvard Medical School, Boston, Massachusetts, United States of America; 4 Division of Pulmonary and Critical Care Medicine, Department of Medicine, Brigham and Women’s Hospital, Boston, Massachusetts, United States of America; The University of Manchester Faculty of Biology Medicine and Health, United Kingdom of Great Britain and Northern Ireland

## Abstract

**Background:**

Fluticasone-salmeterol is available in both metered-dose and dry powder inhaler formulations for the treatment of chronic obstructive pulmonary disease (COPD). Metered-dose inhalers are associated with substantially higher greenhouse gas emissions than dry powder inhalers; however, data on their comparative effectiveness and safety in COPD remain limited. We aimed to compare the effectiveness and safety of fluticasone-salmeterol delivered via metered-dose inhaler (Advair HFA) versus dry powder inhaler (Advair Diskus) among patients with COPD treated in routine care.

**Methods and findings:**

We conducted a retrospective cohort study using Optum’s de-identified Clinformatics DataMart (January 1, 2007 to November 30, 2023). The study included 202,052 commercially insured patients aged 40 years or older with COPD who had continuous insurance coverage for 180 days prior to cohort entry and had not initiated any inhaled corticosteroid–long-acting β₂-agonist during that period. Patients receiving fluticasone-salmeterol via a metered-dose inhaler (exposure) were compared to those receiving these drugs via a dry powder inhaler (referent), with stabilized inverse probability of treatment weighting applied for covariate adjustment. The primary effectiveness outcome was the incidence of first moderate or severe COPD exacerbation within 365 days of cohort entry. The primary safety outcome was the incidence of first pneumonia hospitalization during the same period. Use of fluticasone-salmeterol via metered-dose inhaler was associated with a similar hazard of first moderate or severe COPD exacerbation (hazard ratio [HR], 1.03; 95% confidence interval [CI], 0.99 to 1.08) and first pneumonia hospitalization (HR, 1.06; 95% CI, 0.98 to 1.14) compared to the dry powder inhaler. Primary study limitations include potential residual confounding despite weighting and short follow-up times.

**Conclusions:**

In this cohort study comparing two brand-name fluticasone-salmeterol inhalers prescribed for COPD in routine clinical practice, effectiveness and safety outcomes were similar for patients receiving metered-dose and dry powder versions.

## Author summary

**Why was this study done?:**

- Little real-world evidence exists comparing the effectiveness and safety of metered-dose versus dry powder inhalers for patients with chronic obstructive pulmonary disease (COPD) treated in routine clinical care.
- Health systems around the world have sought to reduce reliance on metered-dose inhalers relative to dry powder inhalers because metered-dose formulations contain propellants that are associated with substantial greenhouse gas emissions.
- Fluticasone-salmeterol is the only brand-name maintenance inhaler therapy currently marketed in the US that is available in both dry powder and metered-dose versions, providing an opportunity to compare clinical outcomes of patients who receive these medications via different delivery devices.

**What did the researchers do and find?:**

- This study analyzed insurance claims data from 202,052 patients with COPD who filled an initial prescription for fluticasone-salmeterol between January 1, 2007, and November 30, 2023.
- A weighting procedure was applied to account for differences in COPD severity, comorbidities, healthcare utilization, and medication use between patients who were prescribed metered-dose versus dry powder fluticasone-salmeterol, helping to reduce potential bias when comparing outcomes between the two groups.
- Patients who received fluticasone-salmeterol via metered-dose inhalers had similar risks of first moderate or severe COPD exacerbation and similar risks of first pneumonia hospitalization compared to patients who received fluticasone-salmeterol via dry powder inhalers.

**What do these findings mean?:**

- As health systems seek to cut down on greenhouse gas emissions by reducing reliance on metered-dose inhalers, this study helps provide further reassurance to patients, clinicians, and payers regarding the effectiveness and safety of fluticasone-salmeterol dry powder inhalers.
- Further studies are needed comparing other metered-dose and dry powder inhalers in asthma and COPD.

## Introduction

The two predominant types of inhalers prescribed to patients with asthma and chronic obstructive pulmonary disease (COPD) are dry powder and metered-dose inhalers [1,2]. Fluticasone-salmeterol, a widely used combined inhaled corticosteroid (ICS)-long-acting β_2_ agonist (LABA), is the only brand-name maintenance therapy for asthma or COPD currently marketed in the US that is available in both dry powder and metered-dose formulations [3]. Health systems around the world have sought to reduce reliance on metered-dose inhalers relative to dry powder inhalers, because metered-dose inhalers contain propellants associated with substantial greenhouse gas emissions [4–7].

Yet, data on the comparative effectiveness and safety of metered-dose versus dry powder inhalers remain limited. One recent study of patients prescribed single-inhaler triple therapy (i.e., a combination of three drugs administered via a single inhaler) in the US found that those who received metered-dose inhalers had a slightly higher risk of moderate or severe COPD exacerbations compared to those who received dry powder inhalers [8]. Similar findings with larger effect sizes were observed in a network meta-analysis of randomized controlled trials in COPD [9]. However, these studies analyzed metered-dose inhalers with active ingredients (budesonide-glycopyrrolate-formoterol) and dosing regimens (twice daily) that differed from the dry powder comparator (once daily fluticasone-umeclidinium-vilanterol).

To date, few studies have directly analyzed the clinical outcomes of patients with COPD receiving identical therapies via different inhaler types. One randomized controlled trial of beclomethasone-glycopyrrolate-formoterol (a product that is not available in the US) found that patients who received therapy via metered-dose inhalers experienced similar effects on forced expiratory volume in 1 second (FEV_1_) at 28 days compared to those who received therapy via dry powder inhalers [10]. Another trial comparing patients who received fluticasone-salmeterol via metered-dose versus dry powder inhaler found similar effects on 28-day FEV_1_ across the two products [11]. These studies, however, were not powered to detect differences in more clinically relevant endpoints such as exacerbations and pneumonia hospitalizations.

A recent matched observational study from the UK that did assess such endpoints found standard-dose fluticasone-salmeterol metered-dose inhalers to be associated with a lower incidence of moderate or severe COPD exacerbations than the equivalent dry powder versions [12]. However, sample sizes were small in this study and investigators matched patients based on a limited set of covariates, raising concern for bias from residual confounding. We therefore performed a new user cohort study in a larger population with more comprehensive covariate-adjustment; the primary aims were to compare the effectiveness and safety of fluticasone-salmeterol delivered via metered-dose inhaler versus dry powder inhaler among patients with COPD treated in routine clinical practice.

## Methods

### Study cohort

This study used data from Optum’s de-identified Clinformatics DataMart, a longitudinal dataset of commercial health insurance and Medicare Advantage enrollees from all 50 US states. Patients were included in the cohort if they had a diagnosis of COPD and initiated an index prescription for fluticasone-salmeterol via a metered-dose inhaler (Advair HFA, exposure) or a dry powder inhaler (Advair Diskus, referent) between January 1, 2007, and November 30, 2023. Initiation of the index prescription marked the cohort entry date. COPD diagnoses were based on International Classification of Diseases, Ninth Revision, Clinical Modification (ICD-9-CM) (491.xx, 492.xx, or 496) and ICD-10-CM (J41.x, J42, J43.x, J44.x) codes; patients were required to have had at least 3 outpatient claims or 1 inpatient claim with these diagnoses in the 3 years before cohort entry (positive predictive value [PPV] 0.82) [13]. We excluded patients younger than 40 years of age to increase the specificity of our COPD definition, and those who did not have continuous insurance coverage during the 180 days before cohort entry. We also excluded patients who filled a prescription for a different ICS-LABA combination inhaler during our baseline assessment period (the 180 days prior to and including the date of cohort entry), as well as patients who filled separate scripts for an ICS and LABA within 30 days of each other during this period. Other maintenance regimens—including monotherapy with an ICS, LABA, or long-acting muscarinic antagonists (LAMA), dual therapy with a LAMA-LABA, or triple therapy with an ICS-LAMA-LABA—were permitted during the baseline assessment period. However, those who initiated another inhaler on the cohort entry date (e.g., a LAMA) in addition to their ICS-LABA were excluded from the analysis since the focus was on patients receiving dual ICS-LABA therapy.

### Assessment of covariates

We measured covariates during a baseline assessment period beginning 180 days prior to and including the date of cohort entry, unless otherwise specified (S1 Fig). The covariates included in our propensity score model were related to COPD severity, comorbidities, healthcare utilization, and medications (see S1 Methods for a complete list of these covariates).

### Outcomes and follow-up

All outcomes were measured beginning the day after cohort entry. The primary effectiveness outcome was time to first moderate or severe COPD exacerbation. A moderate COPD exacerbation was defined as requiring a 5–14-day script for prednisone (PPV 0.73) [14], while a severe COPD exacerbation was defined as requiring hospitalization for COPD (with the following diagnostic codes in the primary position for the hospitalization: ICD-9-CM [491.x, 492.x, or 496] and equivalent codes from the ICD-10-CM [J41.x, J42, J43.x, J44.x] [PPV 0.86]) [15]. We considered events that fit our moderate and severe exacerbation definitions occurring within 14 days of each other as a single severe exacerbation beginning at the time of the earlier exacerbation event.

The primary safety outcome was time to first hospitalization for pneumonia. This was defined as requiring a pneumonia diagnosis in any position using ICD-9-CM codes (PPV 0.88) [16] that were converted to equivalent ICD-10-CM codes based on clinical review (J09.X1, J10.xx-J18.x, A01.03, A02.22, A37.01, A37.11 A37.81, A37.91, A54.84, B01.2, B05.2, B06.81, B77.81, J85.1, J22).

Secondary effectiveness outcomes included the times to first moderate exacerbation and first severe exacerbation (analyzed separately), the annual rate of moderate or severe COPD exacerbations (analyzed jointly), as well as the annual rates of moderate and severe exacerbations (analyzed separately). Secondary safety outcomes included the annual rate of pneumonia hospitalizations and time to death.

Patients were followed for up to 1 year after cohort entry, or until censoring occurred. Reasons for censoring include discontinuation of index therapy (with a 60-day grace period between fills and a 60-day exposure risk window), a switch between treatment groups (exposure to referent or vice versa), dispensing of a different inhaler regiment (ICS, LABA, LAMA, ICS-LABA, LAMA-LABA, ICS-LABA-LABA), death, the end of insurance coverage, the end of data, or November 30, 2023 (whichever occurred earlier).

### Statistical analysis

This study used stabilized inverse probability of treatment weighting (IPTW) to adjust for potential covariate imbalance between the exposure and referent groups. Propensity scores were estimated using an over-identified covariate-balancing propensity score (CBPS) model, which augments standard logistic regression by imposing additional balance constraints, iteratively “nudging” coefficient estimates toward maximizing covariate balance between groups [17]. All covariates in Table 1 were included in the model, and any missing covariate data were marked with missing indicators in our propensity score model. We assessed covariate balance using absolute standardized mean differences between treatment groups, taking an absolute standardized mean difference < 0.1 as indicating an acceptable degree of imbalance for a given covariate [18,19]. Balance was also evaluated for two-way interaction, second degree polynomial, and third degree polynomial terms among covariates [20,21].

**Table 1:** Baseline covariates for new users of fluticasone-salmeterol metered-dose versus dry powder inhalers.

Variable	Advair Diskus (*n* = 177,992)	Advair HFA (*n* = 24,060)	Absolute standardized mean difference	Absolute standardized mean difference after inverse probability of treatment weighting
**Demographics**				
Age, mean (SD), y	68.97 (10.54)	69.78 (10.16)	0.080	0.050
Sex^*^, No. (%)				
Female	103,046 (57.9)	14,733 (61.2)	0.069	0.007
Male	74,901 (42.1)	9,321 (38.7)	0.069	0.007
Unreported or missing	45 (0.0)	6 (0.0)	<0.001	0.001
Race/ethnicity^*^, No. (%)				
Asian	3,239 (1.8)	351 (1.5)	0.030	0.001
Black	21,251 (11.9)	3,184 (13.2)	0.038	0.001
Hispanic	13,355 (7.5)	1,796 (7.5)	0.001	0.002
White	130,320 (73.2)	17,275 (71.8)	0.031	0.003
Unreported or missing	9,827 (5.5)	1,454 (6.0)	0.022	0.001
Region^*^, No. (%)				
Northeast	19,053 (10.7)	2,182 (9.1)	0.057	0.034
Midwest	34,073 (19.1)	4,717 (19.6)	0.012	0.029
South	76,306 (42.9)	10,900 (45.3)	0.049	0.030
West	48,430 (27.2)	6,244 (26.0)	0.029	0.037
Unreported or missing	130 (0.1)	17 (0.1)	0.001	<0.001
**Year of cohort entry, No. (%)**				
2007	14,818 (8.3)	383 (1.6)	0.538	0.027
2008	13,380 (7.5)	575 (2.4)	0.336	0.010
2009	15,260 (8.6)	752 (3.1)	0.313	0.004
2010	15,607 (8.8)	941 (3.9)	0.251	0.005
2011	14,807 (8.3)	1,047 (4.4)	0.194	0.008
2012	15,073 (8.5)	1,202 (5.0)	0.159	0.004
2013	15,756 (8.9)	1,337 (5.6)	0.144	0.001
2014	12,908 (7.3)	1,187 (4.9)	0.107	0.002
2015	12,171 (6.8)	1,385 (5.8)	0.046	<0.001
2016	12,209 (6.9)	1,562 (6.5)	0.015	0.005
2017	13,125 (7.4)	1,666 (6.9)	0.018	0.007
2018	11,543 (6.5)	1,578 (6.6)	0.003	0.007
2019	3,771 (2.1)	1,682 (7.0)	0.191	0.005
2020	1923 (1.1)	1,718 (7.1)	0.235	0.001
2021	1961 (1.1)	2071 (8.6)	0.268	0.004
2022	2023 (1.1)	2,620 (10.9)	0.313	0.004
2023	1,657 (0.9)	2,354 (9.8)	0.298	0.002
**Baseline lung disease**				
Moderate COPD exacerbations, mean (SD)	0.28 (0.60)	0.34 (0.66)	0.084	0.005
Severe COPD exacerbations, mean (SD)	0.08 (0.30)	0.07 (0.29)	0.017	0.010
Pneumonia hospitalizations, mean (SD)	0.09 (0.37)	0.10 (0.38)	0.023	0.004
Any prior claim for asthma, No. (%)	89,337 (50.2)	11,778 (49.0)	0.025	0.005
Respiratory antibiotic fills, mean (SD)	1.04 (1.34)	1.00 (1.35)	0.032	0.006
LAMA, No. (%)	32,106 (18.0)	3,905 (16.2)	0.049	0.006
LABA, No. (%)	1932 (1.1)	217 (0.9)	0.019	0.009
ICS, No. (%)	9,766 (5.5)	1,725 (7.2)	0.065	0.007
LAMA-LABA, No. (%)	892 (0.5)	372 (1.5)	0.085	0.003
ICS-LAMA-LABA, No. (%)	333 (0.2)	433 (1.8)	0.121	<0.001
SABA fills, mean (SD)	1.24 (2.00)	1.35 (2.04)	0.055	0.009
SAMA fills, mean (SD)	0.11 (0.65)	0.08 (0.60)	0.042	0.001
SAMA-SABA fills, mean (SD)	0.43 (1.30)	0.40 (1.22)	0.028	0.004
Long-term azithromycin, No. (%)	754 (0.4)	178 (0.7)	0.037	0.004
Roflumilast, No. (%)	580 (0.3)	108 (0.4)	0.018	<0.001
CPAP or BiPAP, No. (%)	11,319 (6.4)	2017 (8.4)	0.073	<0.001
Spirometry, No. (%)	40,151 (22.6)	5,760 (23.9)	0.032	0.028
Smoking, No. (%)	57,841 (32.5)	10,365 (43.1)	0.214	0.007
Home oxygen or equipment claim, No. (%)	40,388 (22.7)	5,685 (23.6)	0.022	0.002
Pulmonary rehabilitation, No. (%)	869 (0.5)	119 (0.5)	0.001	0.001
Index prescription by pulmonologist, No. (%)	8,357 (4.7)	1,534 (6.4)	0.069	0.008
**Events in the 30 days leading up to cohort entry**				
Moderate or severe COPD exacerbations, No. (%)	27,826 (15.6)	4,277 (17.8)	0.056	<0.001
Respiratory antibiotic fill, No. (%)	47,405 (26.6)	6,051 (25.1)	0.034	0.001
Prednisone fill, No. (%)	12,554 (7.1)	1,567 (6.5)	0.022	0.004
**Other comorbidities**				
Combined comorbidity index, mean (SD)	3.23 (2.96)	3.81 (3.21)	0.179	0.011
Frailty score, mean (SD)	0.21 (0.07)	0.21 (0.07)	0.079	0.015
Obstructive sleep apnea, No. (%)	20,618 (11.6)	4,099 (17.0)	0.145	0.009
Hypertension, No. (%)	121,577 (68.3)	17,208 (71.5)	0.071	0.010
Diabetes, No. (%)	54,021 (30.4)	7,658 (31.8)	0.032	0.007
Obesity, No. (%)	22,088 (12.4)	4,309 (17.9)	0.143	0.015
Coronary artery disease, No. (%)	47,834 (26.9)	6,876 (28.6)	0.038	0.005
Peripheral vascular disease, No. (%)	29,911 (16.8)	4,583 (19.0)	0.057	0.006
Venous thromboembolic disease, No. (%)	6,845 (3.8)	913 (3.8)	0.003	0.003
Congestive heart failure, No. (%)	39,868 (22.4)	5,800 (24.1)	0.040	0.011
GERD, No. (%)	36,636 (20.6)	6,284 (26.1)	0.126	0.012
Renal failure, No. (%)	26,709 (15.0)	3,973 (16.5)	0.041	0.010
Osteoporosis, No. (%)	11,480 (6.4)	1836 (7.6)	0.044	0.005
Dementia/other neurologic disease, No. (%)	7,130 (4.0)	1,400 (5.8)	0.077	0.009
Cancer, nonmetastatic, No. (%)	19,945 (11.2)	2,787 (11.6)	0.012	<0.001
Metastatic solid organ cancer, No. (%)	3,283 (1.8)	538 (2.2)	0.026	0.003
Anxiety disorder, No. (%)	25,168 (14.1)	4,770 (19.8)	0.143	0.009
Depression, No. (%)	26,436 (14.9)	4,571 (19.0)	0.106	0.007
**Baseline healthcare utilization**				
Emergency department visits, mean (SD)	1.54 (3.31)	1.72 (3.09)	0.056	<0.001
Hospitalizations, mean (SD)	0.50 (1.04)	0.53 (1.05)	0.021	0.007
90-day readmissions, mean (SD)	0.12 (0.53)	0.13 (0.55)	0.018	0.004
Office visits, mean (SD)	5.89 (4.92)	6.32 (5.13)	0.084	0.010
Pulmonology office visits, mean (SD)	0.11 (0.52)	0.13 (0.55)	0.050	0.008
Prescription drug claims, mean (SD)	27.79 (20.60)	28.11 (20.64)	0.015	<0.001
BMP or CMP, No. (%)	110,038 (61.8)	16,613 (69.0)	0.156	0.008
CBC with differential, No. (%)	91,929 (51.6)	13,794 (57.3)	0.115	0.013
Electrocardiogram, No. (%)	85,565 (48.1)	12,107 (50.3)	0.045	0.002
Echocardiogram, No. (%)	39,604 (22.3)	5,996 (24.9)	0.062	0.005
CT scan, No. (%)	55,300 (31.1)	8,459 (35.2)	0.086	0.010
Bronchoscopy or biopsy, No. (%)	3,459 (1.9)	504 (2.1)	0.011	0.001
Colonoscopy, No. (%)	1,328 (0.7)	142 (0.6)	0.020	0.005
Mammography, No. (%)	19,020 (10.7)	2,798 (11.6)	0.029	0.004
Bone mineral density scan, No. (%)	7,955 (4.5)	1,129 (4.7)	0.011	0.001
Influenza vaccination, No. (%)	47,131 (26.5)	6,942 (28.9)	0.052	0.014
**Non-pulmonary medications**				
Statins, No. (%)	84,167 (47.3)	12,691 (52.7)	0.109	0.013
β-blockers, No. (%)	62,511 (35.1)	9,268 (38.5)	0.070	0.010
ACEIs, No. (%)	54,247 (30.5)	6,503 (27.0)	0.078	0.010
ARBs, No. (%)	31,022 (17.4)	5,054 (21.0)	0.088	0.003
Calcium-channel blockers, No. (%)	47,132 (26.5)	6,801 (28.3)	0.040	0.010
Thiazide diuretics, No. (%)	39,268 (22.1)	5,024 (20.9)	0.029	0.001
Loop diuretics, No. (%)	43,113 (24.2)	6,034 (25.1)	0.020	0.012
Proton-pump inhibitors, No. (%)	58,601 (32.9)	8,862 (36.8)	0.081	0.008
H_2_-receptor blockers, No. (%)	10,159 (5.7)	1823 (7.6)	0.071	0.004
Metformin, No. (%)	23,371 (13.1)	3,369 (14.0)	0.025	0.001
Sulfonylureas, No. (%)	13,961 (7.8)	1,683 (7.0)	0.033	0.003
SGLT2 inhibitors, No. (%)	763 (0.4)	375 (1.6)	0.091	0.003
DPP-4 inhibitors, No. (%)	4,550 (2.6)	697 (2.9)	0.020	<0.001
GLP-1 receptor agonists, No. (%)	1,760 (1.0)	543 (2.3)	0.085	0.004
Benzodiazepines, No. (%)	30,891 (17.4)	4,361 (18.1)	0.020	0.009
SSRIs/SNRIs, No. (%)	50,615 (28.4)	7,486 (31.1)	0.058	0.014

*Sex, race/ethnicity, and region were the only covariates with unreported or missing values.

LAMA: long-acting muscarinic antagonist; LABA: long-acting beta agonist; ICS: inhaled corticosteroid; SABA: short-acting beta agonist; SAMA: short-acting muscarinic antagonist; CPAP: continuous positive airway pressure; BiPAP: bilevel positive airway pressure; GERD: gastroesophageal reflux disease; BMP: basic metabolic panel; CMP: complete metabolic panel; CBC: complete blood counts; CT: computed tomography; ACEI: angiotensin-converting enzyme inhibitor; ARB: angiotensin-receptor blocker; SGLT2: sodium–glucose co-transporter-2; DPP-4: dipeptidyl peptidase-4; GLP-1: glucagon-like peptide 1; SSRI: selective serotonin reuptake inhibitor; SNRI: serotonin–norepinephrine reuptake inhibitors.

We selected and pre-specified the use of CBPS-based IPTW in our protocol based on diagnostics performed prior to implementation of our study and any evaluation of outcome data. Applying IPTW using a CBPS model resulted in closer covariate balance and less variability in weights in our dataset than other propensity score models we tested (S1 Table). This is consistent with other simulation studies demonstrating strong performance of IPTW with CBPS [22].

We estimated hazard ratios (HRs) and 95% confidence intervals (CIs) using an IPTW-adjusted Cox proportional hazards regression model and robust standard errors (S1 Methods). The target estimand was the average treatment effect across all eligible study participants over on-treatment follow-up (see S2 Table for a complete description of our target trial emulation approach) [23]. Secondary outcomes assessing annual rates of moderate or severe COPD exacerbations and annual rates of pneumonia hospitalizations were estimated using an IPTW-adjusted negative binomial model. The study protocol initially specified use of an adjusted Poisson model to estimate rate outcomes, but this was switched to a negative binomial model to help minimize any effects from overdispersion.

We conducted prespecified exploratory subgroup analyses stratified by whether patients had advanced age (65 years or older), at least 1 baseline moderate or severe exacerbation, at least 1 baseline COPD hospitalization, prior asthma diagnosis codes (both all-time and within the last 3 years, analyzed separately), baseline receipt of spirometry, an index prescription from a pulmonologist, and cohort entry before 2019 (when the first generic version of fluticasone-salmeterol inhalers entered the US market). We performed analyses using data from each subgroup separately (i.e., by fitting separate outcome models to the data in each subgroup of the overall propensity score weighted cohorts).

We conducted a range of prespecified sensitivity analyses to check the robustness of our results. We varied the grace period between prescription fills to 30 and 90 days instead of 60 days and excluded the first 30 and first 60 days after cohort entry when assessing outcomes. We performed an intention-to-treat analysis where patients were followed for the entire year, regardless of whether they discontinued or switched treatments. We also varied several of our outcome measure definitions. We evaluated three alternative definitions of a moderate exacerbation: (1) requiring an office or emergency department visit before the prednisone fill, (2) requiring both a prednisone and a respiratory antibiotic fill on the same day, or (3) permitting either a prednisone or a respiratory antibiotic fill. We also varied the definition of severe exacerbation to permit a COPD exacerbation code in any position, and pneumonia hospitalization to require diagnosis codes in the primary position. We performed 1:1 high-dimensional propensity score (hdPS) matching, an automated algorithm that adjusts for hundreds of empirically defined covariates based on ICD-9 and ICD-10 diagnosis codes and procedure codes for inpatient confinements and medical services, Current Procedural Terminology codes for medical services, and prescription drug claims (based on National Drug Code generic names) [24–26]. In a post-hoc sensitivity analysis, we performed Cox-based estimation of the primary outcomes while treating death as a competing risk using the *causalCmprsk* package in R [27].

Statistical analyses were performed with R, version 4.3.2 (R Foundation for Statistical Computing). IPTW was performed using the *WeightIt* package [28], and covariate balance was assessed using the *cobalt* package [29]. Cox proportional hazards models were performed using the *survival* package and negative binomial models using the *MASS* package [30,31]. Weighted Kaplan–Meier curves were generated using the *adjustedCurves* package [32]. All tests were 2-sided and set at an *α* = 0.05 confidence level. The complete protocol for this study was preregistered at the Center for Open Science Real World Evidence Registry and is included in the Supporting Information (S1 Protocol). This study is reported according to the Strengthening the Reporting of Observational Studies in Epidemiology (STROBE) guidelines (S1 Checklist). The study was approved by the Mass General Brigham Institutional Review Board (2023P000164) with a waiver of informed consent.

## Results

**Fig 1:**
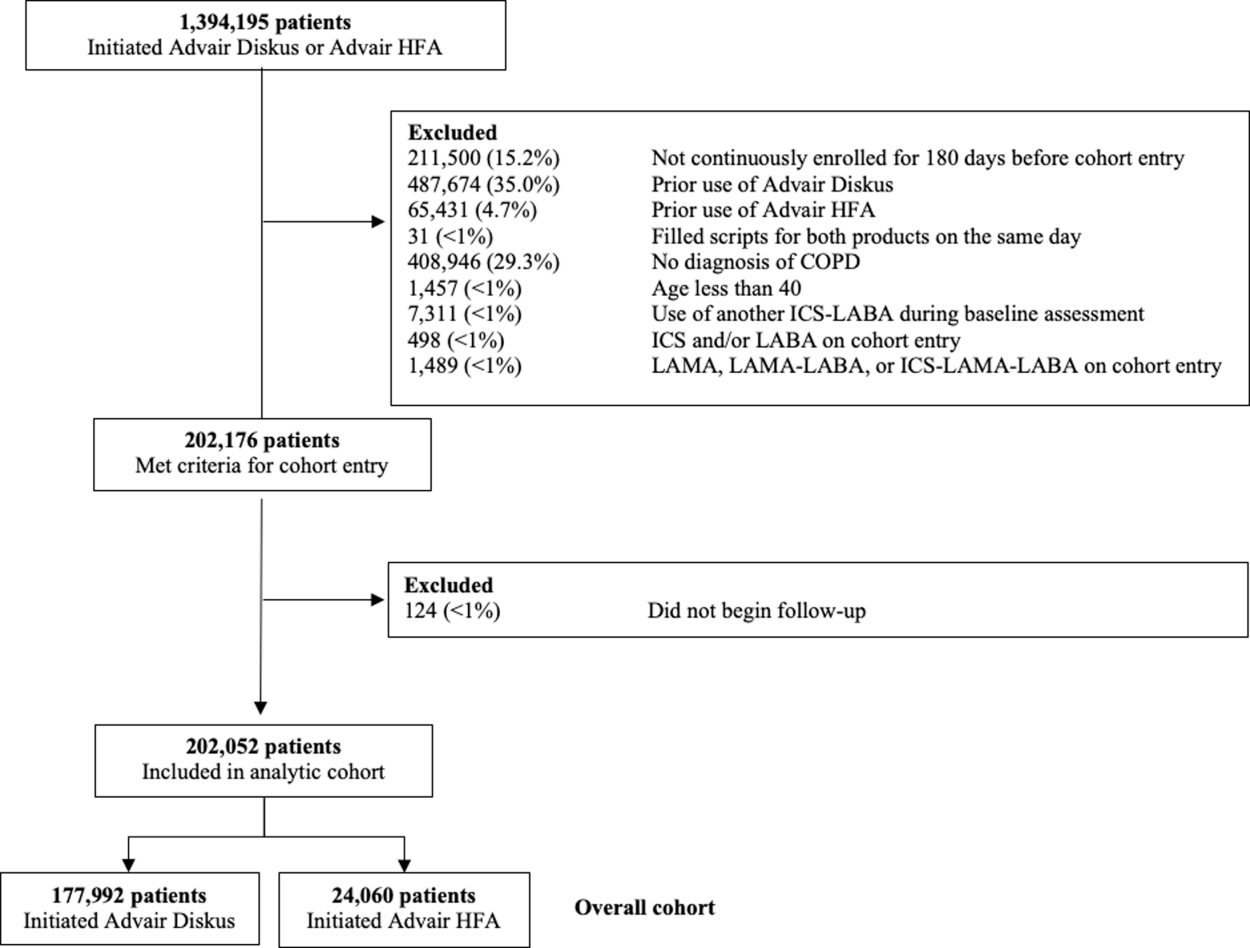
Cohort composition. This graphical representation of cohort composition shows how exclusion criteria were applied prior to cohort entry.

The cohort included 202,052 patients who initiated an index prescription for fluticasone-salmeterol between January 1, 2007 and November 30, 2023; 177,992 patients received fluticasone-salmeterol dry-powder inhalers (referent) and 24,060 received fluticasone-salmeterol metered-dose inhalers (exposure) (Fig 1). The primary outcome measures were the incidence of first moderate or severe COPD exacerbation (effectiveness outcome) and the incidence of first pneumonia hospitalization (safety outcome) in the 365 days following cohort entry.

Patients in the exposure group had slightly more occurrences of moderate COPD exacerbations at baseline (mean, 0.34 [standard deviation {SD}] 0.66 versus 0.28 [SD 0.60]), slightly fewer severe COPD exacerbations (mean, 0.07 [SD 0.29] versus 0.08 [SD 0.30]), and higher comorbidity scores (mean, 3.81 [SD 3.21] versus 3.23 [SD 2.96]) (Table 1). There was greater relative uptake of brand-name fluticasone-salmeterol metered-dose inhalers toward the end of the study period, with a substantially higher proportion of patients who filled their index prescription between 2019 and 2023. After applying IPTW, the groups were well balanced with a mean standardized absolute difference of 0.01 (and all standardized absolute differences substantially below the pre-defined threshold of 0.1) (Table 1). The distribution of propensity scores is shown in S2 Fig.

### Primary effectiveness and safety outcomes

Overall, 30,581 (15.1%) patients experienced a moderate or severe COPD exacerbation. The average hazard of experiencing a first moderate or severe COPD exacerbation was similar for patients who received fluticasone-salmeterol metered-dose inhalers compared to patients who received fluticasone-salmeterol dry powder inhalers (HR, 1.03; 95% CI, 0.99 to 1.08) (Table 2).

**Table 2:** First moderate or severe COPD exacerbation and first pneumonia hospitalization in new users of fluticasone-salmeterol metered-dose versus dry powder inhalers.

Variable	Events per 1,000 person-years		Unadjusted hazard ratio^*^ (95% CI)	Adjusted hazard ratio^*^ (95% CI)
**Advair Diskus**	**Advair HFA**
Moderate or severe COPD exacerbation	419.7	449.2	1.06 (1.02–1.093)	1.03 (0.99–1.08)
Moderate COPD exacerbation	351.5	390.2	1.10 (1.06–1.14)	1.04 (0.99–1.09)
Severe COPD exacerbation	84.9	76.0	0.89 (0.82–0.96)	1.01 (0.92–1.12)
Pneumonia hospitalization	117.1	126.2	1.06 (1.00–1.13)	1.05 (0.97–1.14)

*Hazard ratios greater than 1 mean that patients receiving Advair HFA have a higher hazard of experiencing the relevant outcome.

A total of 9,328 (4.6%) patients in our cohort experienced a pneumonia hospitalization. The hazard of experiencing a first pneumonia hospitalization was similar for patients who received fluticasone-salmeterol metered-dose inhalers compared to patients who received fluticasone-salmeterol dry powder inhalers (HR, 1.05; 95% CI, 0.97 to 1.14).

**Fig 2:**
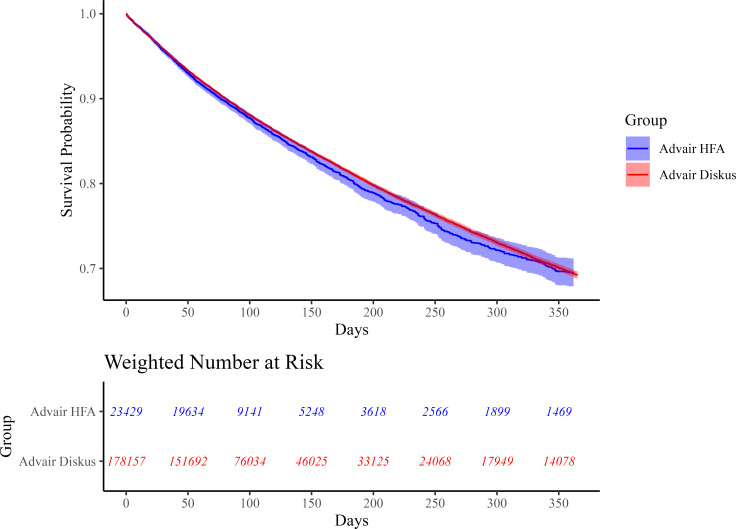
Weighted Kaplan–Meier plot for first moderate or severe COPD exacerbation. This figure shows the probability of experiencing no moderate or severe COPD exacerbation in the 365 days of follow-up for patients in the CBPS-IPTW-adjusted cohort. The blue curve represents new users of Advair HFA (exposure) while the red curve represents new users of Advair Diskus (referent). Bands represent 95% confidence intervals. The proportionality assumption of the Cox model did not appear violated in the primary effectiveness analysis given a null Schoenfeld residual test (*p* = 0.2) and parallel curves on the LML plot (S3 Fig).

**Fig 3:**
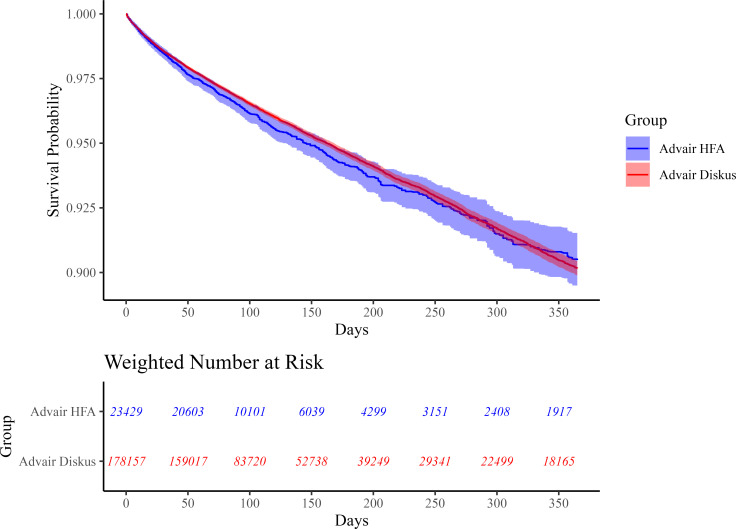
Weighted Kaplan–Meier plot for first pneumonia hospitalization. This figure shows the probability of experiencing no pneumonia hospitalization in the 365 days of follow-up for patients in the CBPS-IPTW-adjusted cohort. The blue curve represents new users of Advair HFA (exposure) while the red curve represents new users of Advair Diskus (referent). Bands represent 95% confidence intervals. The proportionality assumption of the Cox model may not hold for the primary safety analysis given a significant Schoenfeld residual test (*p* = 0.029), though the parallel curves on the LML plot (S4 Fig) suggest that the violation may be minor.

**Fig 4:**
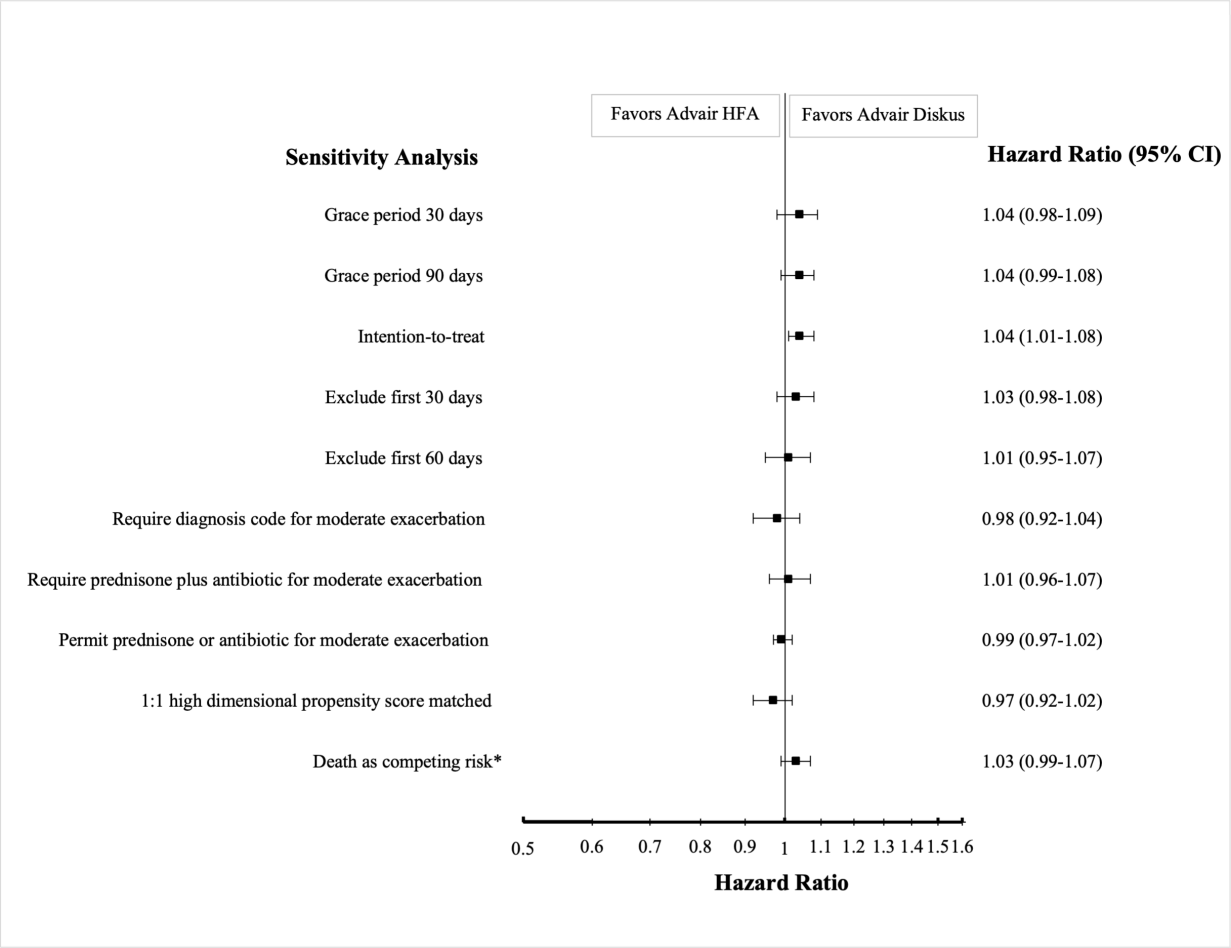
Sensitivity analyses for first moderate or severe COPD exacerbation. This figure shows the hazard ratios and 95% confidence intervals of first moderate or severe COPD exacerbation in new users of fluticasone-salmeterol metered-dose (Advair HFA) versus dry powder (Advair Diskus) inhalers across a range of prespecified sensitivity analyses. Hazard ratios greater than 1 mean that patients receiving fluticasone-salmeterol metered-dose inhalers have a higher hazard of first moderate or severe COPD exacerbation. *The output of the Cox-based model with death as a competing risk is a risk ratio rather than a hazard ratio, with bootstrap-generated confidence intervals.

Median follow-up times were similar for the two groups when analyzing the primary effectiveness outcome (S3 Table) and the primary safety outcome (S4 Table). Weighted Kaplan–Meier curves for the primary effectiveness and safety analyses showed consistent effects over the 365 days of follow-up (see Figs 2 and 3 for these Kaplan–Meier curves and S3 and S4 Figs for testing of the proportionality assumption) [33]. The breakdown of reasons for censoring in the primary effectiveness and safety analyses can be found in S3 and S4 Tables. Sensitivity analyses for the outcomes of first moderate or severe COPD exacerbation (Fig 4) and first pneumonia hospitalization (S5 Fig) yielded results consistent with those of the primary analyses.

### Secondary effectiveness and safety outcomes

When comparing the times to first moderate and severe COPD exacerbation separately, patients who received fluticasone-salmeterol metered-dose inhalers had similar hazards of experiencing a first moderate exacerbation (HR, 1.04; 95% CI: 0.99 to 1.09) and severe exacerbation (HR, 1.01; 95% CI: 0.92 to 1.12) compared to those who received fluticasone-salmeterol dry powder inhalers. The annual rates at which patients experienced either moderate or severe COPD exacerbations (rate ratio (RR), 1.05; 95% CI, 1.01 to 1.10) and the annual rates at which they experienced moderate exacerbations (RR, 1.07; 95% CI, 1.02 to 1.12) were slightly higher among those receiving fluticasone-salmeterol metered-dose inhalers compared to dry powder versions. The annual rates of severe exacerbations (RR, 0.96; 95% CI, 0.87 to 1.07) and pneumonia hospitalizations (RR, 1.07; 95% CI, 0.99 to 1.16), as well as time to all-cause mortality (HR, 1.00; 95% CI: 0.91 to 1.10), were similar between the two treatment groups.

### Subgroup analyses

**Fig 5:**
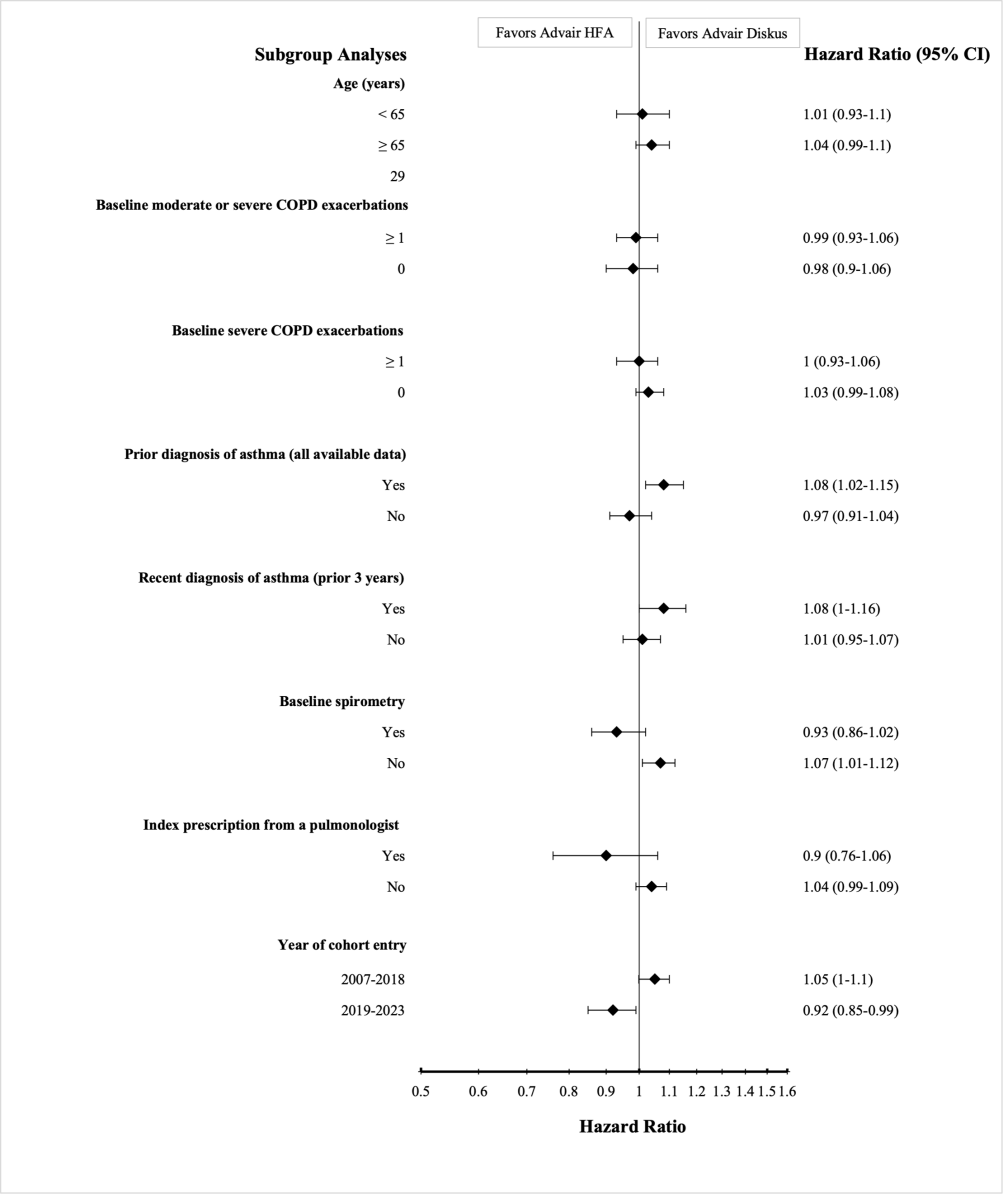
Subgroup analyses for first moderate or severe COPD exacerbation. This figure shows the hazard ratios and 95% confidence intervals of first moderate or severe COPD exacerbation in new users of fluticasone-salmeterol metered-dose (Advair HFA) versus dry powder (Advair Diskus) inhalers across a range of prespecified subgroup analyses. Hazard ratios greater than 1 mean that patients receiving fluticasone-salmeterol metered-dose inhalers have a higher hazard of first moderate or severe COPD exacerbation.

Key exploratory subgroup analyses yielded findings that were similar to our primary analysis. Those receiving metered-dose rather than dry-powder fluticasone-salmeterol had similar risks of moderate or severe COPD exacerbations when restricted to patients with no prior asthma diagnosis codes (HR 0.97, 95% CI 0.91–1.04) and no recent asthma diagnoses (HR 1.01, 95% CI 0.95–1.07) and when cohort entry was restricted to the years (2007−2018) when only brand-name versions of both products were available on the US market (HR 1.05, 95% CI 1.00–1.10) (Fig 5). Similar findings were observed on subgroup analyses of pneumonia hospitalizations (S6 Fig).

## Discussion

Use of fluticasone-salmeterol via metered-dose inhalers was associated with a similar hazard of first moderate or severe COPD exacerbation and a similar hazard of first pneumonia hospitalization compared to use fluticasone-salmeterol via dry powder inhalers in a cohort of patients with COPD treated in routine clinical practice. Similar clinical outcomes were observed when analyzing key secondary endpoints and across a range of prespecified sensitivity and subgroup analyses. These findings may help provide reassurance to health systems seeking to reduce their reliance on metered-dose inhalers that dry powder formulations of fluticasone-salmeterol are associated with similar clinical outcomes as metered-dose versions.

Our findings are consistent with a prior study showing no difference in pneumonia risk between real-world users of metered-dose and dry powder fluticasone-salmeterol inhalers [12]. But, unlike that study, we did not detect differences in the risk of first moderate or severe COPD exacerbation across these products; our study relied on a substantially larger sample size (202,052 versus 1,684 patients) and adjusted for a larger set of relevant covariates (80 versus 6), potentially reducing bias. Our findings add to a growing body of literature showing similar outcomes for patients with COPD receiving metered-dose versus dry powder inhalers.

Although ICS-LABAs have long been prescribed as a first-line dual therapy in COPD, this class of medications now occupies a less prominent role in consensus clinical guidelines [1]. Following the pivotal FLAME clinical trial published in 2016, which found increased rates of both COPD exacerbations and pneumonia hospitalizations in patients prescribed fluticasone-salmeterol rather than indacaterol-glycopyrronium [34], the Global Initiative for Chronic Obstructive Lung Disease (GOLD) shifted to favor LAMA-LABAs over ICS-LABAs for most patients with COPD in their updated 2017 guidelines [35]. In 2023, GOLD recommendations shifted again to favor ICS-LAMA-LABAs over ICS-LABAs for patients requiring an inhaled corticosteroid, such as those with frequent exacerbations, asthma, and/or high eosinophil levels [36].

Nevertheless, comparing metered-dose and dry powder ICS-LABAs remains important for several reasons. First and most importantly, these inhalers are still widely prescribed in COPD despite shifts in clinical guidelines. Additionally, unlike brand-name fluticasone-salmeterol metered-dose inhalers, dry powder versions have faced independent generic competition since 2019. Although our study involved a brand-to-brand comparison, earlier work has found nearly identical clinical outcomes when comparing generic versus brand-name fluticasone-salmeterol dry powder inhalers in patients with COPD [37]. Fluticasone-salmeterol dry powder inhalers may therefore represent a less expensive alternative to metered-dose versions for patients in the US receiving ICS-LABA therapy for COPD.

Second, patients can now receive generic triple therapy (ICS-LAMA-LABA) via two separate inhalers (an ICS-LABA and a LAMA) following the approval of the first generic LAMA in 2023 (tiotropium) [38]. Single-inhaler triple therapy, by contrast, will likely remain on-patent and without generics in the US through the early 2030s [3,39]. The GOLD guidelines recommend that physicians take into account considerations such as cost and ease of use when selecting single- versus dual-inhaler triple therapy [1]. Over the next several years, physicians may therefore confront the question of whether to prescribe more expensive brand-name single-inhaler triple therapy or potentially less expensive generic triple therapy via two separate inhalers. Further studies are needed comparing such regimens in routine clinical practice, but for physicians who elect to prescribe dual-inhaler triple therapy, our study finds no evidence that a dry powder fluticasone-salmeterol is associated with worse clinical outcomes than the metered-dose version.

Third, our findings also contribute to the emerging literature on potential intraclass differences among ICS-containing inhalers in COPD. Prior research has suggested an increased pneumonia risk and, in some cases, a higher exacerbation risk for fluticasone-containing inhalers compared to budesonide-containing inhalers in COPD, whether among patients receiving dual ICS-LABA or triple ICS-LAMA-LABA therapy [40–44]. However, many of these studies did not control for inhaler type (metered-dose versus dry powder), and questions have been raised about whether differences might exist between fluticasone-based therapies administered via different delivery systems. We found no such differences among new users of fluticasone-salmeterol in COPD. Further head-to-head studies are needed comparing budesonide-based versus fluticasone-based therapies administered via different delivery systems in COPD.

Hydrofluoroalkane (HFA)-134a, a common propellant in metered-dose inhalers like fluticasone-salmeterol, has a global warming potential 1,430 times greater than carbon dioxide, contributing to significantly higher greenhouse gas emissions compared to dry powder inhalers [45,46]. Specifically, lifecycle analyses suggest that emissions from metered-dose inhalers are approximately 20 times greater than those of their counterparts that do not use propellants, such as dry powder and soft-mist inhalers [47]. Despite these environmental concerns, metered-dose inhalers still account for the vast majority of all prescribed inhalers in the US, with over 100 million units dispensed annually (with emissions equivalent to 550,000 gas-fueled cars driven for one year) [4,46,48,49]. Several countries have begun focusing on reducing the use of metered-dose inhalers. In England, for example, where metered dose inhalers produce an estimated 800,000 metric tons of annual carbon dioxide equivalent emissions (equivalent to 157,885 US homes’ annual electricity use), the National Health Service has begun encouraging the switching of patients from metered-dose inhalers to propellant-free alternatives [6,50,51]. Comparative effectiveness and safety data for these inhaler types, such as those provided by this study, are therefore valuable in reassuring health systems that similar environmental sustainability goals can be pursued while still ensuring that patients with chronic lung disease receive optimal care.

It is important to note that most metered-dose inhalers dispensed in the US are albuterol rescue inhalers rather than daily maintenance therapies like fluticasone-salmeterol. Thus, although decreased use of fluticasone-salmeterol metered-dose inhalers may be justified based on our results, it would still constitute only a small step towards reduced greenhouse gas emissions. A broader range of initiatives may be needed for health systems seeking to lower their inhaler-related carbon footprint, including efforts to improve control of asthma and COPD and ensure that only patients with documented obstructive lung disease receive inhalers in the first place [52]. In our study, fewer than a quarter of patients had spirometry in the 6 months prior to cohort entry.

Some patients, of course, will also require metered-dose inhalers because of poor peak inspiratory flow; others may have a strong preference for metered-dose inhalers. Thus, efforts to facilitate greater use of dry powder inhalers compared to metered-dose inhalers must be implemented with caution. However, most patients with COPD are capable of using either type of inhaler [53,54]. As such and given that we found no significant differences in clinical outcomes among patients with COPD receiving fluticasone-salmeterol via metered-dose versus dry powder inhalers after controlling for a broad array of covariates, preferential prescribing of the dry powder version may be warranted as a more environmentally sustainable and cost-effective option.

### Limitations

First, although we controlled for numerous covariates, there may still have been some unobserved confounding. One key source of potential confounding is peak inspiratory flow, which could have driven physicians to prescribe metered-dose inhalers to patients with worse lung function (for whom dry powder inhalers may not be suitable and who are more likely to experience exacerbations). But past work has suggested that patients prescribed metered-dose inhalers in clinical practice are only slightly more likely to have suboptimal peak inspiratory flow and that peak inspiratory may not be a limiting factor for most patients with COPD in using dry powder inhalers [53,54]. Further research is needed to understand use patterns and clinical outcomes among patients with poor peak inspiratory flow who receive metered-dose versus dry powder inhalers. Another key source of potential confounding is the ability of patients to use proper technique when administering their inhaler, which could have driven physicians to prescribe dry powder inhalers to patients who struggle more with compliance due to the relative simplicity of the device. To help mitigate the potential impact of these and other unobserved confounders, we controlled for many other factors associated with exacerbations, including prior moderate or severe COPD exacerbations during the baseline assessment period, as well as validated comorbidity [55] and frailty indexes [56]. Differences in the labelling of the two products could also have contributed to confounding; fluticasone-salmeterol dry powder inhalers have a labeled indication for both COPD and asthma in the US, while the metered-dose version only has a labelled indication for asthma, although it is frequently prescribed off-label in COPD. Physicians could have prescribed the metered-dose inhaler more frequently to those with asthma-COPD overlap, and these patients may have been more prone to exacerbations. However, subgroup analyses of patients with no recorded asthma diagnoses (using all available data) and of patients with no recent asthma diagnoses found nearly identical rates of exacerbations between the two treatment groups.

A second set of limitations relates to the short follow-up time in our study. Most patients discontinued therapy soon after initiation, restricting our observation period. In addition, because follow-up was limited to one year, we did not assess long-term outcomes. Third, we observed relatively few pneumonia hospitalizations in our cohort, somewhat limiting the power of our comparative safety analysis. Fourth, we lacked sufficient data on several subgroups of clinical interest, including those with high eosinophils, true asthma-COPD overlap syndrome, and documented reversibility on spirometry, as well as those receiving inhalers off-label. Fifth, use of administrative claims data can be associated with miscoding and measurement error that could potentially bias our results. Sixth, we lacked data on whether patients used their devices with proper technique. Finally, our study population is limited to commercially insured US patients. Although the database contains millions of enrollees across all 50 states, replication of our analyses in other patient populations would be valuable.

In conclusion, patients with COPD who were prescribed fluticasone-salmeterol via metered-dose versus dry powder inhalers were observed to have similar risks of COPD exacerbations and pneumonia hospitalizations in this national insurance claims database study. As health systems seek to reduce their reliance on metered-dose inhalers in favor of dry powder formulations in an effort to reduce greenhouse gas emissions, studies like this one can provide reassurance to patients, clinicians, and payers regarding the safety and effectiveness of lower-emission dry powder inhalers.

## Supporting information

S1 Checklist Strengthening the reporting of observational studies in epidemiology (STROBE) checklist. (DOCX)

S1 Protocol Prospective protocol and analysis plan. (DOCX)

S1 Methods XXX. (DOCX)

S1 Table Relative performance of inverse probability of treatment weighting between selected propensity score models in balancing covariates. a. For every covariate *X*_*i*_, the distributions of their quadratic and cubic transformations (i.e., *X*_*i*_^2^ and *X*_*i*_^3^) as well as their two-way interactions with other covariates (i.e., *X*_i_*X_*j*_) were compared between the exposure and referent groups using standardized mean differences, and the maximum standardized mean difference (i.e., imbalance) among these higher-order terms is reported for each propensity score model. The aim was to balance on higher-order covariate moments and interactions to help achieve optimal balance in observational studies [20,21]. CBPS: covariate-balancing propensity score; GBM: generalized boosted model; GLM: generalized linear model (logistic regression).
(DOCX)

S2 Table Target trial emulation protocol. This table outlines the design elements of a target trial emulation, comparing the idealized design of a randomized controlled trial (target trial) to the corresponding observational emulation conducted using real-world data. The purpose is to align observational study methods with the principles of causal inference in a transparent fashion, highlighting key assumptions, design features, and analytical approaches to estimate causal effects [23]. a. A more detailed description of the statistical analysis can be found in the corresponding paper. LAMA: long-acting muscarinic antagonist; ICS: inhaled corticosteroid; LABA: long-acting beta agonist; CBPS: covariate-balancing propensity score; IPTW: inverse probability of treatment weighting.
(DOCX)

S3 Table Reasons for censoring in the analysis of first moderate or severe COPD exacerbation. a. Median follow-up time in the group of patients receiving Advair Diskus was 88 days (interquartile range [IQR] 88–156 days). Mean follow-up time was 132 days (standard deviation [SD] 96 days). b. Median follow-up time in the group of patients receiving Advair HFA was 88 days (IQR 72–148 days). Mean follow-up time was 120 days (SD 90 days). LAMA: long-acting muscarinic antagonist; ICS: inhaled corticosteroid; LABA: long-acting beta agonist.
(DOCX)

S4 Table Reasons for censoring in the analysis of first pneumonia hospitalization. a. Median follow-up time in the group of patients receiving Advair Diskus was 88 days (interquartile range [IQR] 88–177 days). Mean follow-up time was 144 days (standard deviation [SD] 100 days). b. Median follow-up time in the group of patients receiving Advair HFA was 88 days (IQR 88–155 days). Mean follow-up time was 131 days (SD 95 days). LABA: long-acting beta agonist; LAMA: long-acting muscarinic antagonist; ICS: inhaled corticosteroid.
(DOCX)

S1 Fig Study design comparing new users of Advair HFA and Advair Diskus. This graphical representation of study design shows how exclusion criteria were applied prior to cohort entry and how covariates were assessed [1]. *COPD diagnoses were based on 1 inpatient claim or 3 outpatient claims in the 3 years before cohort entry. **This includes all covariates except events within 30 days, asthma diagnosis codes, and smoking diagnosis codes. ***Events within 30 days include COPD exacerbations, fills of respiratory antibiotics, and fills of prednisone. ICS: inhaled corticosteroid; LABA: long-acting beta-2 agonist.
(TIFF)

S2 Fig Propensity score distributions between new users of Advair HFA and Advair Diskus users. These figures show propensity score distributions between the exposure (Advair HFA) and referent (Advair Diskus) groups before and after adjustment with inverse probability weighting using a covariate-balancing propensity score (CBPS) model. The blue plot captures the propensity score distribution of new users of Advair HFA, the red plot captures the propensity score distribution of new users of Advair Diskus, and purple indicates overlap between the two distributions. The pre-weighting c-statistic was calculated to be 0.766 and the post-weighting c-statistic was calculated to be 0.527.
(TIFF)

S3 Fig Log-minus-log plot for Cox proportional hazards model testing first moderate or severe exacerbation. This figure shows the log-minus-log plot of the Cox proportional hazards model for first moderate or severe COPD exacerbation in new users of fluticasone-salmeterol metered-dose (Advair HFA) versus dry powder (Advair Diskus) inhalers. Parallel curves imply a constant hazard ratio and provides evidence that supports the proportional hazard assumption of the Cox model [33].
(TIFF)

S4 Fig Log-minus-log plot for Cox proportional hazards model testing first pneumonia hospitalization. This figure shows the log-minus-log plot of the Cox proportional hazards model for first pneumonia hospitalization in new users of fluticasone-salmeterol metered-dose (Advair HFA) versus dry powder (Advair Diskus) inhalers. Parallel curves imply a constant hazard ratio and provides evidence that supports the proportional hazard assumption of the Cox model [33].
(TIFF)

S5 Fig Sensitivity analyses for first pneumonia hospitalization. This figure shows the hazard ratios and 95% confidence intervals of first pneumonia hospitalization in new users of fluticasone-salmeterol metered-dose (Advair HFA) versus dry powder (Advair Diskus) inhalers across a range of prespecified sensitivity analyses. Hazard ratios greater than 1 mean that patients receiving fluticasone-salmeterol metered-dose inhalers have a higher hazard of first pneumonia hospitalization. *The output of the Cox-based model with death as a competing risk is a risk ratio rather than a hazard ratio, with bootstrap-generated confidence intervals.
(TIFF)

S6 Fig Subgroup analyses for first pneumonia hospitalization. This figure shows the hazard ratios and 95% confidence intervals of first pneumonia hospitalization in new users of fluticasone-salmeterol metered-dose (Advair HFA) versus dry powder (Advair Diskus) inhalers across a range of prespecified subgroup analyses. Hazard ratios greater than 1 mean that patients receiving fluticasone-salmeterol metered-dose inhalers have a higher hazard of first pneumonia hospitalization.
(TIFF)

## Abbreviations

CBPS: covariate-balancing propensity score
CIs: confidence intervals
COPD: chronic obstructive pulmonary disease
hdPS: high-dimensional propensity score
HFA: hydrofluoroalkane
HRs: hazard ratios
ICS: inhaled corticosteroid
IPTW: inverse probability of treatment weighting
IQR: interquartile range
LABA: long-acting β_2_ agonist
PPV: positive predictive value
