# Supplementary material for: The comparative effectiveness and safety of fluticasone-salmeterol via metered-dose versus dry powder inhalers for COPD: A new user cohort study
Source: PLoS Med. 2025 May 14;22(5):e1004596. doi: 10.1371/journal.pmed.1004596 (PMC12077913; doi:10.1371/journal.pmed.1004596)
Supplement: S1 Checklist — (DOCX) [file pmed.1004596.s001.docx]

**Supporting Information: S1 Checklist**

STROBE Statement—checklist of items that should be included in reports of observational studies

|  | Item No. | Recommendation | Section (Paragraph No.) | Relevant text from manuscript |
| --- | --- | --- | --- | --- |
| **Title and abstract** | 1 | (*a*) Indicate the study’s design with a commonly used term in the title or the abstract | Title page (1) | Title includes the term ‘new user cohort study’ |
|  |  | (*b*) Provide in the abstract an informative and balanced summary of what was done and what was found | Abstract (1-4) | Subsections: Background; Methods and Findings; Conclusions |
| Introduction | | | |  |
| Background/rationale | 2 | Explain the scientific background and rationale for the investigation being reported | Introduction (1-4) | Paragraphs 1-3 entirely focus on scientific and domain background.  “We therefore performed a new user cohort study in a larger population with more comprehensive covariate-adjustment.” |
| Objectives | 3 | State specific objectives, including any prespecified hypotheses | Introduction (4) | The “primary aims were to compare the effectiveness and safety of fluticasone-salmeterol delivered via metered-dose inhaler vs. dry powder inhaler among patients with COPD treated in routine clinical practice.” |
| Methods | | | |  |
| Study design | 4 | Present key elements of study design early in the paper | Methods (1) | Subsection: Study Cohort |
| Setting | 5 | Describe the setting, locations, and relevant dates, including periods of recruitment, exposure, follow-up, and data collection | Methods (1, 3, 6) | Subsections: Study Cohort; Outcomes and Follow-up |
| Participants | 6 | (*a*) *Cohort study*—Give the eligibility criteria, and the sources and methods of selection of participants. Describe methods of follow-up | Methods (1, 3, 6) | Subsections: Study Cohort; Outcomes and Follow-up |
|  |  | (*b*) *Cohort study*—For matched studies, give matching criteria and number of exposed and unexposed | NA | NA |
| Variables | 7 | Clearly define all outcomes, exposures, predictors, potential confounders, and effect modifiers. Give diagnostic criteria, if applicable | Methods (1-5) | Subsections: Study Cohort; Assessment of Covariates Outcomes and Follow-up |
| Data sources/ measurement | 8* | For each variable of interest, give sources of data and details of methods of assessment (measurement). Describe comparability of assessment methods if there is more than one group | Methods (1-5) | Subsections: Study Cohort; Assessment of Covariates; Outcomes and Follow-up |
| Bias | 9 | Describe any efforts to address potential sources of bias | Methods (7, 8) | Subsection: Statistical Analysis  “This study used stabilized inverse probability of treatment weighting (IPTW) to adjust for potential covariate imbalance between the exposure and referent groups.” |
| Study size | 10 | Explain how the study size was arrived at | Figure 1 | Figure 1 is a participant flow diagram |

| Quantitative variables | 11 | Explain how quantitative variables were handled in the analyses. If applicable, describe which groupings were chosen and why | Methods (2-5, 7-9, 11) | Subsections: Assessment of Covariates; Outcomes and Follow-up; Statistical Analysis |
| --- | --- | --- | --- | --- |
| Statistical methods | 12 | (*a*) Describe all statistical methods, including those used to control for confounding | Methods (7-12) | Subsection: Statistical Analysis |
|  |  | (*b*) Describe any methods used to examine subgroups and interactions | Methods (10) | Entire paragraph 10 |
|  |  | (*c*) Explain how missing data were addressed | Methods (7) | “All covariates in Table 1 were included in the model, and any missing covariate data were marked with missing indicators in our propensity score model.” |
|  |  | (*d*) *Cohort study*—If applicable, explain how loss to follow-up was addressed | Methods (6) | Entire paragraph 6 |
|  |  | (*e*) Describe any sensitivity analyses | Methods (11) | Entire paragraph 11 |
| Results | | | | |
| Participants | 13* | (a) Report numbers of individuals at each stage of study—eg numbers potentially eligible, examined for eligibility, confirmed eligible, included in the study, completing follow-up, and analysed | Figure 1 | Figure 1 is a participant flow diagram |
|  |  | (b) Give reasons for non-participation at each stage |  |  |
|  |  | (c) Consider use of a flow diagram |  |  |
| Descriptive data | 14* | (a) Give characteristics of study participants (eg demographic, clinical, social) and information on exposures and potential confounders | Table 1 | Baseline covariates table |
|  |  | (b) Indicate number of participants with missing data for each variable of interest |  |  |
|  |  | (c) *Cohort study*—Summarise follow-up time (eg, average and total amount) | Results (4) | “Median follow-up times were similar for the two groups when analyzing the primary effectiveness outcome (S3 Table) and the primary safety outcome (S4 Table).” |
| Outcome data | 15* | *Cohort study*—Report numbers of outcome events or summary measures over time | Results (2, 3) | “Overall, 30,581 (15.1%) patients experienced a moderate or severe COPD exacerbation.”  “A total of 9,328 (4.6%) patients in our cohort experienced a pneumonia hospitalization.” |
| Main results | 16 | (*a*) Give unadjusted estimates and, if applicable, confounder-adjusted estimates and their precision (eg, 95% confidence interval). Make clear which confounders were adjusted for and why they were included | Table 2, Methods (2) | Table 2 presents the unadjusted hazard ratios for our primary analyses.  The entire “Assessment of Covariates” subsection details which confounders were adjusted for and why. |
|  |  | (*b*) Report category boundaries when continuous variables were categorized | Methods (10) | “We conducted prespecified exploratory subgroup analyses stratified by whether the patient had advanced age (65 years or older), at least 1 baseline moderate or severe exacerbation, at least 1 baseline COPD hospitalization, prior asthma diagnosis codes (both all-time and within the last 3 years, analyzed separately), baseline receipt of spirometry, an index prescription from a pulmonologist, and cohort entry before 2019 (when the first generic version of fluticasone-salmeterol inhalers had entered the US market).” |
|  |  | (*c*) If relevant, consider translating estimates of relative risk into absolute risk for a meaningful time period | Results (4) | We do not explicitly report absolute risk given it would vary based on covariates, but present it visually through a Kaplan-Meier curve:  “Weighted Kaplan-Meier curves for the primary effectiveness and safety analyses showed consistent effects over the 365 days of follow-up (see Figs 2 and 3 for these Kaplan-Meier curves and S3 Figure and S4 Figure for testing of the proportionality assumption).” |

| Other analyses | 17 | Report other analyses done—eg analyses of subgroups and interactions, and sensitivity analyses | Results (4, 6) | “Sensitivity analyses for the outcomes of first moderate or severe COPD exacerbation (Fig 4) and first pneumonia hospitalization (S5 Figure) yielded results consistent with those of the primary analyses.”  Entire “Subgroup Analyses” subsection. |
| --- | --- | --- | --- | --- |
| Discussion | | | | |
| Key results | 18 | Summarise key results with reference to study objectives | Discussion (1) | Entire paragraph 1. |
| Limitations | 19 | Discuss limitations of the study, taking into account sources of potential bias or imprecision. Discuss both direction and magnitude of any potential bias | Discussion (10, 11) | Entire “Limitations” subsection. |
| Interpretation | 20 | Give a cautious overall interpretation of results considering objectives, limitations, multiplicity of analyses, results from similar studies, and other relevant evidence | Discussion (2, 12) | Entire paragraph 2.  “In conclusion, patients with COPD who were prescribed fluticasone-salmeterol via metered-dose vs. dry powder inhalers were observed to have similar risks of COPD exacerbations and pneumonia hospitalizations in this national insurance claims database study.” |
| Generalisability | 21 | Discuss the generalisability (external validity) of the study results | Discussion (8, 9) | Entire paragraphs 8 and 9. |
| Other information | |  | | |
| Funding | 22 | Give the source of funding and the role of the funders for the present study and, if applicable, for the original study on which the present article is based | Funding | Part of additional information provided with submission. |

*Give information separately for exposed and unexposed groups.

**Note:** An Explanation and Elaboration article discusses each checklist item and gives methodological background and published examples of transparent reporting. The STROBE checklist is best used in conjunction with this article (freely available on the Web sites of PLoS Medicine at http://www.plosmedicine.org/, Annals of Internal Medicine at

http://www.annals.org/, and Epidemiology at http://www.epidem.com/). Information on the STROBE Initiative is available at http://www.strobe-statement.org.
