# Supplementary material for: The comparative effectiveness and safety of fluticasone-salmeterol via metered-dose versus dry powder inhalers for COPD: A new user cohort study
Source: PLoS Med. 2025 May 14;22(5):e1004596. doi: 10.1371/journal.pmed.1004596 (PMC12077913; doi:10.1371/journal.pmed.1004596)
Supplement: S1 Protocol — (DOCX) [file pmed.1004596.s002.docx]

**Study Protocol**

**March 4, 2024**

**The comparative effectiveness and safety of fluticasone-salmeterol via metered-dose vs. dry powder inhalers in COPD**

Brandon Demkowicz^1,2^

Kevin Rader, PhD^1^

Shirley Wang, PhD^2,3^

Aaron S. Kesselheim, MD, JD, MPH^2,3^

William B. Feldman, MD, DPhil, MPH^2,3,4^

^1^ Harvard University, Department of Statistics

^2^ Division of Pharmacoepidemiology and Pharmacoeconomics, Department of Medicine, Brigham and Women’s Hospital

^3^ Harvard Medical School

^4^ Division of Pulmonary and Critical Care Medicine, Department of Medicine, Brigham and Women’s Hospital

**Correspondence to**: Dr. Feldman, Division of Pharmacoepidemiology and Pharmacoeconomics, Department of Medicine, Brigham and Women’s Hospital, 1 Brigham Circle, Boston MA 02120, 617-278-0930, [wbfeldman@bwh.harvard.edu](mailto:wbfeldman@bwh.harvard.edu)

**Funding:** This work was funded by a grant (National Heart, Lung, and Blood Institute K08HL163246) to Dr. Feldman.

**Disclosures**: Dr. Feldman serves as a consultant for Alosa Health and as an expert witness in litigation against inhaler manufacturers. He previously served as a consultant for Aetion. The funders had no role in the design and conduct of the study; collection, management, analysis, interpretation, of the data; review, or approval of the manuscript; and decision to submit the manuscript for publication.

**Part 1: Background**

Fluticasone-salmeterol has long been approved for the treatment of chronic obstructive pulmonary disease (COPD). In the US, GlaxoSmithKline markets two different inhalers containing fluticasone-salmeterol: Advair Diskus (first approved in 2000) and Advair HFA (first approved in 2006). The former, a dry powder inhaler, is approved for the indications of both COPD and asthma, while the latter, a metered-dose inhaler, is only approved for the indication of asthma. However, a small randomized controlled trial showed that Advair HFA had similar efficacy to Advair Diskus when treating COPD.^1^ Yet, metered dose inhalers have also been associated with more errors than dry powder inhalers.

Given ongoing clinical uncertainty, we will analyze the comparative effectiveness and safety of fluticasone-salmeterol delivered via metered-dose inhaler (Advair HFA) vs dry powder inhaler (Advair Diskus) among patients with COPD treated in routine clinical practice. The primary effectiveness outcome will be first moderate or severe COPD exacerbation; the primary safety outcome will be first pneumonia hospitalization. Secondary outcomes will include the annual rate of total moderate or severe COPD exacerbations (analyzed separately and together), first moderate exacerbation, first severe exacerbation, and annual rate of total pneumonia hospitalizations.

**Part 2: Protocol**

Protocol Version Date: 3/4/2024

Author(s): Brandon Demkowicz, Kevin Rader, Shirley Wang, Aaron S. Kesselheim, William B. Feldman

1. **Title:** The comparative effectiveness and safety of fluticasone-salmeterol via metered-dose vs. dry powder inhalers in COPD
2. **Core Research Team:** Brandon, Kevin, Shirley, Aaron, Will (Implementation in the Aetion Evidence Platform: Will; PI: Dr. William Feldman)
3. **Aim(s)**

**Aim 1 (effectiveness):** To compare new users of fluticasone-salmeterol via metered-dose vs. dry powder inhalers on the outcome of first moderate or severe COPD exacerbation.

**Aim 2 (safety)**: To compare new users of fluticasone-salmeterol via metered-dose vs. dry powder inhalers on the outcome of first pneumonia hospitalization.

1. **Data source**: Optum Clinformatics claims database January 1, 2007-November 30, 2023. The study will begin in 2007 since that is the first full year after Advair HFA was approved (Advair Diskus was approved in 2000). Thus, both products were on the US market throughout the study period.
2. **Study Design Diagram:** see Appendix Figure 1
3. **Cohort Identification**

**6.1 Cohort Summary**

All patients with COPD, defined by 3 outpatient claims or 1 inpatient claim in the prior 3 years (PPV 0.82 [0.72-0.89]),^2^ who initiated a prescription for fluticasone-salmeterol (Advair HFA) (exposure) vs. fluticasone-salmeterol (Advair Diskus) (referent) between January 1, 2007 and November 30, 2023. We will require a 180-day washout period for both the exposure and the referent. We will also require 180 days of continuous insurance coverage prior to cohort entry. We will include patients who received other therapy in the baseline period (whether single-agent inhalers [ICS, LAMA, LABA], LAMA-LABAs, or triple therapy). In other words, we will include patients who may be stepping up therapy (from monotherapy), stepping down therapy (triple therapy), or switching (from LAMA-LABA). Following other studies, we will exclude patients who are less than age 40 to increase the specificity of COPD diagnoses. This will help further ensure that patients entering the cohort are receiving triple therapy for COPD rather than another respiratory condition.

**6.2 Important steps for cohort creation**

***Cohort entry date:*** The cohort entry date is of the first claim for fluticasone-salmeterol (Advair HFA) (exposure) vs. fluticasone-salmeterol (Advair Diskus) (referent) between January 1, 2007 and November 30, 2023

***Study period:***

Baseline Period: The baseline period is defined as 180 days prior and up to and including the cohort entry date [-180, 0] unless otherwise noted in the design diagram.

Follow up Period: The follow-up period starts 1 day after the cohort entry date and continues until censoring. For further details on censoring, see section 8.2.

***Note:*** All diagnosis, procedure and generic names of drugs are given in the file named “Protocol Appendix.”

| Steps |  |
| --- | --- |
| **6.2.1** | **Patients with prescription for Advair HFA/Diskus**  *Advair HFA is the exposure and Advair Diskus is the reference* **(fills of both on cohort entry are not allowed)**  **Date of prescription is called the index date**  [assessed between January 1, 2007 and November 30,2023]  *Appendix Table 1 “Exposure definitions,” variables names: Advair HFA, Advair Diskus* |
| **6.2.2** | **Exclude patients who do not have continuous insurance enrollment during baseline period**  [assessed between 180 days prior up to and including index date (-180, 0)] |
| **6.2.3** | **Exclude patients with no prior diagnosis of COPD**  [assessed 3 years prior to and including the index date (-1095, 0)]  *Appendix Table 2 “Exclusion criteria,” variable name: Diagnosis of COPD* |
| **6.2.4** | **Exclude patients who are under the age of 40**  [assessed on the index date (0, 0)] |
| **6.2.5** | **Exclude patients who received another ICS-LABA script (besides Advair HFA or Advair Diskus)**  [assessed between 180 days prior up to and including index date (-180, 0)  *Appendix Table 2 “Exclusion criteria,” variable name: ICS-LABA besides Advair* |
| **6.2.6** | **Exclude patients who started a LAMA, LAMA-LABA, or ICS-LAMA-LABA on the cohort entry date**  [assessed on the cohort entry date (0,0)] |

1. **Covariates**

The baseline covariate assessment period for all measures will start 180 days before cohort entry and will stretch up to and include the day of cohort entry with the following exceptions: (1) We will assess age, sex, race, region, year of cohort entry, and index prescription by a pulmonologist only on the day of cohort entry; (2) We will assess the presence of any prior asthma diagnosis using all available data and stretching up to (and including) the day of cohort entry. Diagnosis based covariates will be defined using any care setting and any diagnosis position unless otherwise specified. All codes for these variables are provided in Appendix Table 3, “Covariates.” The variable names are as listed below in Table 1. All variables listed below will be included in our propensity score model. We will use a missing indicator in our propensity score model in the case that data are missing for any covariates.

**Table 1: Patient characteristics**

| **Characteristic** |
| --- |
|  |
| **Demographics** |
| Age |
| Female |
| Race |
| Region |
| **Year of cohort entry** |
| **Baseline lung disease** |
| Moderate COPD exacerbations |
| Severe COPD exacerbations |
| Total COPD exacerbations (moderate or severe) |
| SABA fills |
| SAMA fills |
| SAMA-SABA fills |
| Pneumonia hospitalizations |
| Respiratory antibiotic fills^b^ |
| Prior diagnosis of asthma (all available data) |
| Claim for home oxygen or oxygen equipment |
| CPAP/BiPAP |
| Spirometry |
| Index prescription by pulmonologist |
| Smoking |
| Pulmonary rehabilitation |
| LAMA |
| LABA |
| ICS |
| LAMA-LABA |
| ICS-LAMA-LABA |
| Chronic azithromycin |
| Roflumilast |
| Chronic prednisone |
| **Events within 30 days of cohort entry** |
| Moderate or severe COPD exacerbations |
| Respiratory antibiotic fill^b^ |
| **Other co-morbidities** |
| Combined comorbidity index |
| Frailty score |
| Obstructive sleep apnea |
| Hypertension |
| Diabetes |
| Obesity |
| Coronary artery disease |
| Peripheral vascular disease |
| Venous thromboembolic disease (DVT or PE) |
| Congestive heart failure |
| GERD |
| Renal failure |
| Osteoporosis |
| Dementia/other neurologic disease |
| Malignancy, non-metastatic |
| Metastatic solid organ malignancy |
| Anxiety disorder |
| Depression |
| **Healthcare utilization in baseline period** |
| Emergency department visits |
| Hospitalizations |
| 90-day readmissions |
| Office visits |
| Pulmonologist visits |
| Prescription drug claims |
| BMP or CMP |
| CBC with differential ordered |
| Electrocardiogram |
| Echocardiogram |
| CT scan |
| Bronchoscopy |
| Colonoscopy |
| Mammography |
| Bone-mineral density scan |
| Influenza vaccination |
| **Non-pulmonary medications** |
| Statins |
| Beta-blockers |
| ACE-inhibitors |
| ARBs |
| Calcium channel blockers |
| Thiazide-like and thiazide-type diuretics |
| Loop diuretics |
| Proton-pump inhibitors |
| H2-receptor blockers |
| Metformin |
| Sulfonylureas |
| SGLT2 inhibitors |
| DPP-4 inhibitors |
| GLP-1 receptor agonists |
| Benzodiazepines |
| SSRIs or SNRIs |

a. Our method for identifying respiratory antibiotics is described in Appendix Table 4, “Method for identifying respiratory antibiotics.

1. Outcome variable and study follow-up
   1. Outcome variables and study follow-up:

**Primary effectiveness outcome:** First moderate or severe COPD exacerbation

Moderate COPD exacerbations are defined as requiring prednisone for 5-14 days, and severe COPD exacerbations are defined as requiring hospitalization (with COPD in the primary position). The full codes for moderate exacerbations, severe exacerbations, and moderate or severe exacerbations are below. Defining moderate exacerbations based on prednisone fills (of 5-14 days) has been validated with PPV of 0.73 (0.70-0.77).^3^ Note that, on sensitivity analysis, we will require both a prednisone and an antibiotic fill (PPV 0.80 [95% CI 0.74-0.86]).^3^ Defining severe COPD exacerbations based on COPD diagnosis codes in the primary position has been validated with a PPV of 0.86.^4^

The primary analysis is time to first moderate or severe COPD exacerbation. In secondary analyses (see below), we will analyze cumulative exacerbations during one year of follow up using a Prenctice, Williams, and Peterson Total Time Model. We will require that distinct episodes of exacerbation be separated by at least 14 days. Therefore, if a patient meets the criteria for an exacerbation more than once within 14 days, this will be counted as 1 unique episode that begins at the first exacerbation within the episode.

Episodes that meet both moderate and severe exacerbation criteria within 14 days of each other will be counted as 1 severe exacerbation that begins at the time of meeting the criteria for the first exacerbation within the episode, regardless of whether the criteria are for a moderate or severe exacerbation.

Therefore, there are 2 types of prednisone fills that would otherwise meet criteria for moderate exacerbations but are instead grouped as severe COPD exacerbations: (1) First, prednisone fills that occurred within 14 days AFTER the end of a COPD hospitalization will count as a severe COPD exacerbation beginning with the start of the COPD hospitalization. The prednisone script is likely just completing the therapy that was begun in the hospital and so should not count as a new exacerbation. (2) Second, prednisone scripts that occurred within 14 days BEFORE the start of a COPD hospitalization will count as a severe COPD exacerbation beginning with the start of the prednisone script. When prednisone is not enough and hospitalization is required, this episode is a severe exacerbation.

**8.1.A. Moderate exacerbation**

**1. New Sequence**

- 1.1. The occurrence of [**Continuous prednisone (14D)**](https://bwh-dope.aetion.com/measures/details/603159/1495/0/basics), starting at least one day after the cohort entry date; and
- 1.2. The occurrence of [**COPD hospitalization (COPD codes, primary)**](https://bwh-dope.aetion.com/measures/details/603148/1495/0/basics), not occurring within 14 days before the start of 1.1, and until 14 days after the end of 1.1.

The event of interest in this group is 1.1.

NOTE: Below are further definitions of terms used to construct the moderate exacerbation definition.

[**Continuous**](https://bwh-dope.aetion.com/measures/details/603159/1495/0/basics) **prednisone (14D) is is defined as:**

[**Prednisone fill**](https://bwh-dope.aetion.com/measures/details/603156/1495/0/basics), with an allowed gap of 14 days and an episode extension of 0 days.

**COPD hospitalization (COPD codes, primary)** is defined as:

- The occurrence of **Inpatient Confinement** with the following attributes:
  - **Diagnosis Code (Primary Position), ICD-9** is any of: { “491”, “491.0”, “491.1”, “491.21”, “491.22”, “491.8”, “491.9”, “492”, “492.0”, “492.8”, “496” }
    - 491 - CHRONIC BRONCHITIS
    - 491.0 - SIMPLE CHRONIC BRONCHITIS
    - 491.1 - MUCOPURULENT CHRONIC BRONCHITIS
    - 491.21 - OBSTRUCTIVE CHRONIC BRONCHITIS WITH (ACUTE) EXACERBATION
    - 491.22 - OBSTRUCTIVE CHRONIC BRONCHITIS WITH ACUTE BRONCHITIS
    - 491.8 - OTHER CHRONIC BRONCHITIS
    - 491.9 - UNSPECIFIED CHRONIC BRONCHITIS
    - 492 - EMPHYSEMA
    - 492.0 - EMPHYSEMATOUS BLEB
    - 492.8 - OTHER EMPHYSEMA
    - 496 - CHRONIC AIRWAY OBSTRUCTION NOT ELSEWHERE CLASSIFIED
- The occurrence of **Inpatient Confinement** with the following attributes:
  - **Diagnosis Code (Primary Position), ICD-10** is any of: { “J41.0”, “J41.1”, “J41.8”, “J43.0”, “J43.1”, “J43.2”, “J43.8”, “J43.9”, “J44.0”, “J44.1”, “J44.9”, “J42”, “J41”, “J43”, “J44” }
    - J41.0 - Simple chronic bronchitis
    - J41.1 - Mucopurulent chronic bronchitis
    - J41.8 - Mixed simple and mucopurulent chronic bronchitis
    - J43.0 - Unilateral pulmonary emphysema [MacLeod's syndrome]
    - J43.1 - Panlobular emphysema
    - J43.2 - Centrilobular emphysema
    - J43.8 - Other emphysema
    - J43.9 - Emphysema, unspecified
    - J44.0 - Chronic obstructive pulmonary disease with acute lower respiratory infection
    - J44.1 - Chronic obstructive pulmonary disease with (acute) exacerbation
    - J44.9 - Chronic obstructive pulmonary disease, unspecified
    - J42 - Unspecified chronic bronchitis
    - J41 - Simple and mucopurulent chronic bronchitis
    - J43 - Emphysema
    - J44 - Other chronic obstructive pulmonary disease

**8.1.B. Severe COPD exacerbation**

This measure identifies continuous days that the following measure has:

[**Severe COPD exacerbation (14D, COPD codes primary) w/out specifying allowable gap /w severe episodes**](https://bwh-dope.aetion.com/measures/details/603154/1495/0/basics), with an allowed gap of 14 days and an episode extension of 0 days.

This measure creates a flag that is true on the days that are identified:

- [**COPD hospitalization and NO continuous prednisone in prior 14 days (COPD codes primary)**](https://bwh-dope.aetion.com/measures/details/603152/1495/0/basics)
- [**COPD hospitalization w/in 14D of continuous prednisone (COPD codes, primary)**](https://bwh-dope.aetion.com/measures/details/603151/1495/0/basics)

NOTE: Below are further definitions of terms used to construct the moderate exacerbation definition.

- [**COPD hospitalization and NO continuous prednisone in prior 14 days (COPD codes primary)**](https://bwh-dope.aetion.com/measures/details/603152/1495/0/basics)

This measure was defined as:

**1. New Sequence**

- 1.1. The occurrence of [**COPD hospitalization (COPD codes, primary)**](https://bwh-dope.aetion.com/measures/details/603148/1495/0/basics); and
- 1.2. The occurrence of [**Continuous prednisone (14D)**](https://bwh-dope.aetion.com/measures/details/603159/1495/0/basics), not occurring within 14 days before the start of 1.1, and until 0 days before the start of 1.1.

The event of interest in this group is 1.1.

- [**COPD hospitalization w/in 14D of continuous prednisone (COPD codes, primary)**](https://bwh-dope.aetion.com/measures/details/603151/1495/0/basics)

This measure was defined as:

**1. New Sequence**

- 1.1. The occurrence of [**Continuous prednisone (14D)**](https://bwh-dope.aetion.com/measures/details/603159/1495/0/basics); and
- 1.2. The occurrence of [**COPD hospitalization (COPD codes, primary)**](https://bwh-dope.aetion.com/measures/details/603148/1495/0/basics), starting within 0 days after the start of 1.1, and until 14 days after the end of 1.1.

The event of interest in this group is 1.1.

**8.1.C. Moderate or Severe COPD Exacerbation**

This measure creates a flag that is true on the days that are identified:

- [**Moderate COPD exacerbation (14D, COPD codes primary)**](https://bwh-dope.aetion.com/measures/details/603153/1495/0/basics) as defined in 8.1.A above.
- [**Severe COPD exacerbation (14D, COPD codes primary) specifying 14D allowable gap**](https://bwh-dope.aetion.com/measures/details/603160/1495/0/basics) as defined in 8.1.B. above.

**Primary safety outcome: First hospitalization for pneumonia**

We will allow a pneumonia diagnosis in any position in our prior work since this is an acute condition (and we can be confident that, if somebody has a pneumonia diagnosis during a hospitalization, he/she is experiencing an acute process—which stands in contrast to a COPD diagnosis in any position during the hospitalization, where we cannot be sure that this is an acute process). The full codes for pneumonia hospitalizations are below and have been validated with a PPV of 0.88 (95% CI 0.82-0.92)^5^ using ICD-9 codes. We converted ICD-9 codes to ICD-10 codes using clinical review, because our study period was after the transition to using ICD-10 codes in the United States.

This event measure was defined as occurring when any of the following criteria were met:

- The occurrence of **Inpatient Confinement** with the following attributes:
  - **Diagnosis Code (Any Confinement Position), ICD-9** is any of: { “480”, “480.0”, “480.1”, “480.2”, “480.3”, “480.8”, “480.9”, “481”, “482”, “482.0”, “482.1”, “482.2”, “482.3”, “482.30”, “482.31”, “482.32”, “482.39”, “482.4”, “482.40”, “482.41”, “482.42”, “482.49”, “482.8”, “482.81”, “482.82”, “482.83”, “482.84”, “482.89”, “482.9”, “483”, “483.0”, “483.1”, “483.8”, “484”, “484.1”, “484.3”, “484.5”, “484.6”, “484.7”, “484.8”, “485”, “486”, “487.0”, “488.01”, “488.11”, “488.81” }
    - 480 - VIRAL PNEUMONIA
    - 480.0 - PNEUMONIA DUE TO ADENOVIRUS
    - 480.1 - PNEUMONIA DUE TO RESPIRATORY SYNCYTIAL VIRUS
    - 480.2 - PNEUMONIA DUE TO PARAINFLUENZA VIRUS
    - 480.3 - PNEUMONIA DUE TO SARS-ASSOCIATED CORONAVIRUS
    - 480.8 - PNEUMONIA DUE TO OTHER VIRUS NOT ELSEWHERE CLASSIFIED
    - 480.9 - VIRAL PNEUMONIA UNSPECIFIED
    - 481 - PNEUMOCOCCAL PNEUMONIA [STREPTOCOCCUS PNEUMONIAE PNEUMONIA]
    - 482 - OTHER BACTERIAL PNEUMONIA
    - 482.0 - PNEUMONIA DUE TO KLEBSIELLA PNEUMONIAE
    - 482.1 - PNEUMONIA DUE TO PSEUDOMONAS
    - 482.2 - PNEUMONIA DUE TO HEMOPHILUS INFLUENZAE (H. INFLUENZAE)
    - 482.3 - PNEUMONIA DUE TO STREPTOCOCCUS
    - 482.30 - PNEUMONIA DUE TO STREPTOCOCCUS UNSPECIFIED
    - 482.31 - PNEUMONIA DUE TO STREPTOCOCCUS GROUP A
    - 482.32 - PNEUMONIA DUE TO STREPTOCOCCUS GROUP B
    - 482.39 - PNEUMONIA DUE TO OTHER STREPTOCOCCUS
    - 482.4 - PNEUMONIA DUE TO STAPHYLOCOCCUS
    - 482.40 - PNEUMONIA DUE TO STAPHYLOCOCCUS UNSPECIFIED
    - 482.41 - METHICILLIN SUSCEPTIBLE PNEUMONIA DUE TO STAPHYLOCOCCUS AUREUS
    - 482.42 - METHICILLIN RESISTANT PNEUMONIA DUE TO STAPHYLOCOCCUS AUREUS
    - 482.49 - OTHER STAPHYLOCOCCUS PNEUMONIA
    - 482.8 - PNEUMONIA DUE TO OTHER SPECIFIED BACTERIA
    - 482.81 - PNEUMONIA DUE TO ANAEROBES
    - 482.82 - PNEUMONIA DUE TO ESCHERICHIA COLI [E.COLI]
    - 482.83 - PNEUMONIA DUE TO OTHER GRAM-NEGATIVE BACTERIA
    - 482.84 - PNEUMONIA DUE TO LEGIONNAIRES' DISEASE
    - 482.89 - PNEUMONIA DUE TO OTHER SPECIFIED BACTERIA
    - 482.9 - BACTERIAL PNEUMONIA UNSPECIFIED
    - 483 - PNEUMONIA DUE TO OTHER SPECIFIED ORGANISM
    - 483.0 - PNEUMONIA DUE TO MYCOPLASMA PNEUMONIAE
    - 483.1 - PNEUMONIA DUE TO CHLAMYDIA
    - 483.8 - PNEUMONIA DUE TO OTHER SPECIFIED ORGANISM
    - 484 - PNEUMONIA IN INFECTIOUS DISEASES CLASSIFIED ELSEWHERE
    - 484.1 - PNEUMONIA IN CYTOMEGALIC INCLUSION DISEASE
    - 484.3 - PNEUMONIA IN WHOOPING COUGH
    - 484.5 - PNEUMONIA IN ANTHRAX
    - 484.6 - PNEUMONIA IN ASPERGILLOSIS
    - 484.7 - PNEUMONIA IN OTHER SYSTEMIC MYCOSES
    - 484.8 - PNEUMONIA IN OTHER INFECTIOUS DISEASES CLASSIFIED ELSEWHERE
    - 485 - BRONCHOPNEUMONIA ORGANISM UNSPECIFIED
    - 486 - PNEUMONIA ORGANISM UNSPECIFIED
    - 487.0 - INFLUENZA WITH PNEUMONIA
    - 488.01 - INFLUENZA DUE TO IDENTIFIED AVIAN INFLUENZA VIRUS WITH PNEUMONIA
    - 488.11 - INFLUENZA DUE TO IDENTIFIED 2009 H1N1 INFLUENZA VIRUS WITH PNEUMONIA
    - 488.81 - INFLUENZA DUE TO IDENTIFIED NOVEL INFLUENZA A VIRUS WITH PNEUMONIA
- The occurrence of **Inpatient Confinement** with the following attributes:
  - **Diagnosis Code (Any Confinement Position), ICD-10** is any of: { “J09.X1”, “J10.0”, “J10.00”, “J10.01”, “J10.08”, “J11.0”, “J11.00”, “J11.08”, “J12”, “J12.0”, “J12.1”, “J12.2”, “J12.3”, “J12.8”, “J12.81”, “J12.89”, “J12.9”, “J13”, “J14”, “J15”, “J15.0”, “J15.1”, “J15.2”, “J15.20”, “J15.21”, “J15.211”, “J15.212”, “J15.29”, “J15.3”, “J15.4”, “J15.5”, “J15.6”, “J15.7”, “J15.8”, “J15.9”, “J16”, “J16.0”, “J16.8”, “J17”, “J18”, “J18.0”, “J18.1”, “J18.2”, “J18.8”, “J18.9”, “A01.03”, “A02.22”, “A37.01”, “A37.11”, “A37.81”, “A37.91”, “A54.84”, “B01.2”, “B05.2”, “B06.81”, “B77.81”, “J85.1”, “J22” }
    - J09.X1 - Influenza due to identified novel influenza A virus with pneumonia
    - J10.0 - Influenza due to other identified influenza virus with pneumonia
    - J10.00 - Influenza due to other identified influenza virus with unspecified type of pneumonia
    - J10.01 - Influenza due to other identified influenza virus with the same other identified influenza virus pneumonia
    - J10.08 - Influenza due to other identified influenza virus with other specified pneumonia
    - J11.0 - Influenza due to unidentified influenza virus with pneumonia
    - J11.00 - Influenza due to unidentified influenza virus with unspecified type of pneumonia
    - J11.08 - Influenza due to unidentified influenza virus with specified pneumonia
    - J12 - Viral pneumonia, not elsewhere classified
    - J12.0 - Adenoviral pneumonia
    - J12.1 - Respiratory syncytial virus pneumonia
    - J12.2 - Parainfluenza virus pneumonia
    - J12.3 - Human metapneumovirus pneumonia
    - J12.8 - Other viral pneumonia
    - J12.81 - Pneumonia due to SARS-associated coronavirus
    - J12.89 - Other viral pneumonia
    - J12.9 - Viral pneumonia, unspecified
    - J13 - Pneumonia due to Streptococcus pneumoniae
    - J14 - Pneumonia due to Hemophilus influenzae
    - J15 - Bacterial pneumonia, not elsewhere classified
    - J15.0 - Pneumonia due to Klebsiella pneumoniae
    - J15.1 - Pneumonia due to Pseudomonas
    - J15.2 - Pneumonia due to staphylococcus
    - J15.20 - Pneumonia due to staphylococcus, unspecified
    - J15.21 - Pneumonia due to staphylococcus aureus
    - J15.211 - Pneumonia due to Methicillin susceptible Staphylococcus aureus
    - J15.212 - Pneumonia due to Methicillin resistant Staphylococcus aureus
    - J15.29 - Pneumonia due to other staphylococcus
    - J15.3 - Pneumonia due to streptococcus, group B
    - J15.4 - Pneumonia due to other streptococci
    - J15.5 - Pneumonia due to Escherichia coli
    - J15.6 - Pneumonia due to other Gram-negative bacteria
    - J15.7 - Pneumonia due to Mycoplasma pneumoniae
    - J15.8 - Pneumonia due to other specified bacteria
    - J15.9 - Unspecified bacterial pneumonia
    - J16 - Pneumonia due to other infectious organisms, not elsewhere classified
    - J16.0 - Chlamydial pneumonia
    - J16.8 - Pneumonia due to other specified infectious organisms
    - J17 - Pneumonia in diseases classified elsewhere
    - J18 - Pneumonia, unspecified organism
    - J18.0 - Bronchopneumonia, unspecified organism
    - J18.1 - Lobar pneumonia, unspecified organism
    - J18.2 - Hypostatic pneumonia, unspecified organism
    - J18.8 - Other pneumonia, unspecified organism
    - J18.9 - Pneumonia, unspecified organism
    - A01.03 - Typhoid pneumonia
    - A02.22 - Salmonella pneumonia
    - A37.01 - Whooping cough due to Bordetella pertussis with pneumonia
    - A37.11 - Whooping cough due to Bordetella parapertussis with pneumonia
    - A37.81 - Whooping cough due to other Bordetella species with pneumonia
    - A37.91 - Whooping cough, unspecified species with pneumonia
    - A54.84 - Gonococcal pneumonia
    - B01.2 - Varicella pneumonia
    - B05.2 - Measles complicated by pneumonia
    - B06.81 - Rubella pneumonia
    - B77.81 - Ascariasis pneumonia
    - J85.1 - Abscess of lung with pneumonia
    - J22 - Unspecified acute lower respiratory infection

**Secondary**

1. All-cause mortality
2. Annual rate of moderate or severe COPD exacerbations
3. Annual rate of moderate COPD exacerbations
4. Annual rate of severe COPD exacerbations
5. First moderate COPD exacerbation
6. First severe COPD exacerbation
7. Annual rate of hospitalizations for pneumonia
   1. Follow up and censoring

**Primary effectiveness and safety outcomes**: Follow up starts 1 day after the cohort entry date and continues until any of the following occurs:

(1) discontinuation of index therapy (with a 60-day grace period between fills and a 60-day exposure risk window)

(2) switch from exposure to referent or vice-versa

(3) initiation of a different inhaler regimen (ICS, LABA, ICS-LABA, LAMA-LABA, ICS-LABA-LABA)

(4) death

(5) the end of insurance coverage

(6) the end of data

(7) 365 days

1. **Statistical analysis**: For our primary balancing methodology, we will perform inverse probability of treatment weighting (IPTW) with stabilized weights using propensity scores estimated from an over-identified covariate balancing propensity score (CBPS) model. We specified a logistic regression model for the CBPS estimation procedure, including the variables in Table 1 as predictors of exposure.

CBPS augments standard GLM propensity score estimation by imposing additional balance constraints, optimizing the likelihood function while iteratively “nudging” coefficient estimates toward a model that maximizes covariate balance between groups.^6,7^ This method has been shown to offer increased robustness to propensity score model misspecification, improved covariate balance in observational data, and reduced bias in estimating treatment effects compared to logistic regression, while still maintaining its ease of interpretability.^6,8^ IPTW is a standard method for estimating the average treatment effect (ATE), and the use of stabilized weights can offer increased precision.^9–11^ Furthermore, we found that CBPS-IPTW performed the best in our particular dataset in terms of achieving strong balance while avoiding extreme weights that could bias our estimates.^12^ This balance assessment is summarized in Table 2, which compares several methods on a set of important balance metrics.^7,13,14^ While entropy weighting outperformed in several important balance metrics, it made less efficient use of the data with a lower effective sample size (ESS) in the exposure group and also had extremely large weights that could bias our estimates. Several other metrics were also computed for both the main effect and higher order terms, such as the maximum standardized mean difference (SMD), the maximum Kolmogorov-Smirnov (KS) for higher order terms, the number of variance ratios greater than 0.5 and less than 2, the number of SMDs for higher order terms that exceeded 0.1, and finally, the ESS, weight ratio, and coefficient of variability (CV) for the referent group. However, these metrics were comparable between all the methods, and were therefore excluded from Table 2.

**
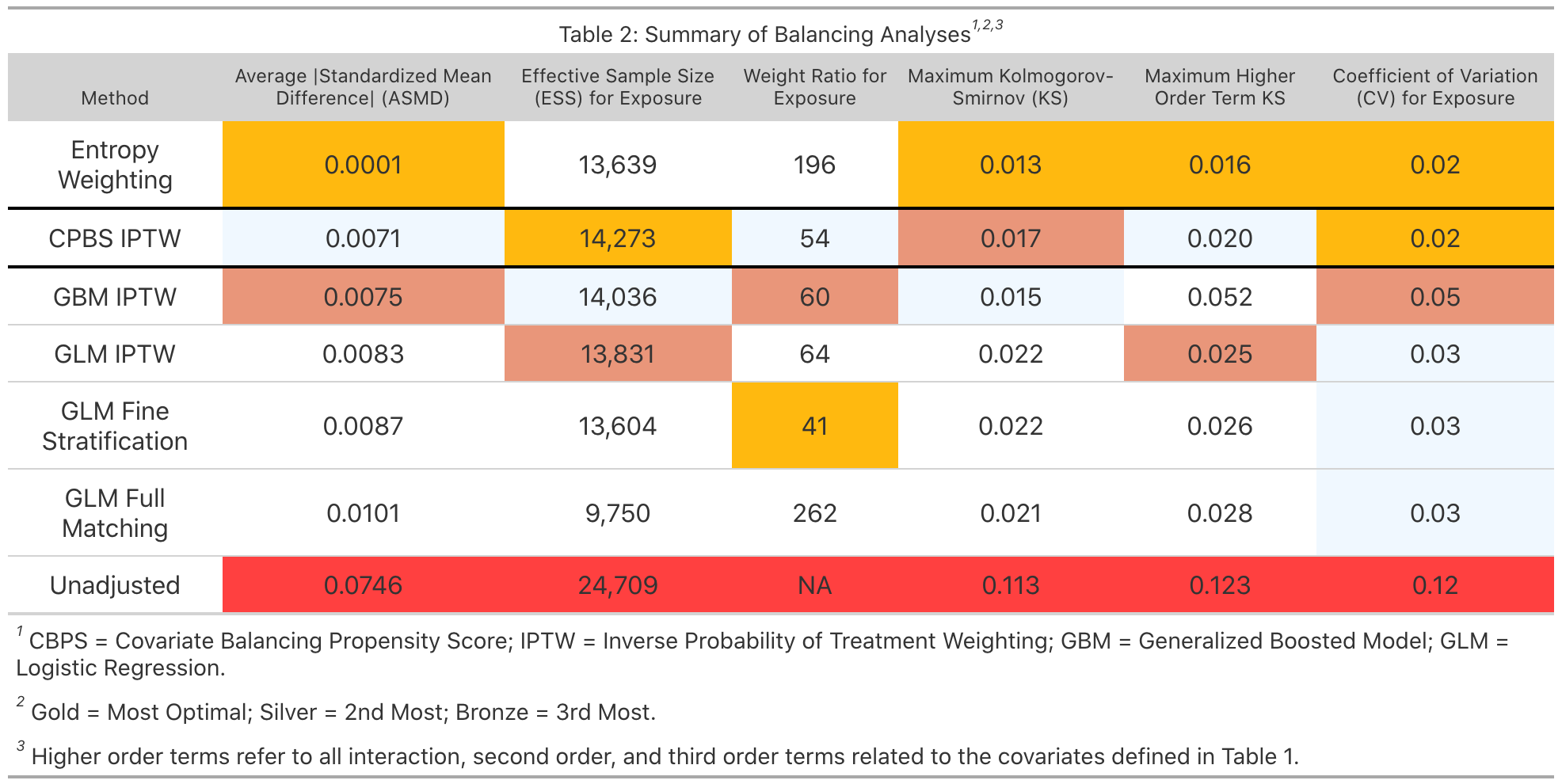
**

To estimate the relative average treatment effect (ATE) on our time-to-event outcomes (including the primary effectiveness and safety outcomes, as well as our secondary outcomes of moderate COPD exacerbation and severe COPD exacerbation), we will estimate marginal hazard ratios (HRs) using Cox proportional hazards models of the respective outcome with the treatment as the sole predictor and incorporating balancing weights from CBPS-IPTW.^10,12^ The corresponding 95% confidence intervals will be estimated using cluster-robust standard errors (SEs).^12^ To estimate the ATE on annual rates of recurrent time-to-event outcomes (our secondary outcomes of annual rates of COPD exacerbations and pneumonia hospitalizations), we will use a Prentice, Williams, and Peterson Total Time Model in the weighted cohort, with a 14-day washout period after each exacerbation. This modified version of the Cox model does not assume constant baseline risk or independence between recurrent outcomes for a given individual, which is consistent with what we know about the increasing risk of subsequent COPD exacerbations.^15–19^ We will report both unadjusted and adjusted results for primary and secondary outcomes. In addition to hazard ratios, we will present the number of outcomes, mean duration of follow up, and reasons for censoring stratified by exposure. We will also produce adjusted cumulative incidence curves for both primary outcomes.^20,21^

1. **Subgroup analyses**

|  | **Subgroup*** | **Why?** | **Strengths** | **Weaknesses** |
| --- | --- | --- | --- | --- |
| **1** | 1 or more baseline moderate or severe COPD exacerbations | ICS-based regimens are thought to be especially valuable for those who frequently exacerbate. This subgroup analysis seeks to examine the comparative effectiveness of Advair Diskus and HFA in this population where ICS-based regimens are thought to be particularly beneficial. | Addresses comparative effectiveness and safety in high-risk patients | None |
| **2** | 1 or more baseline COPD hospitalizations | ICS-based regimens are thought to be especially valuable for those who frequently exacerbate. This subgroup analysis seeks to examine the comparative effectiveness of Advair Diskus and HFA in this population where ICS-based regimens are thought to be particularly beneficial. | Addresses comparative effectiveness and safety in high-risk patients | Low power |
| **3** | Prior diagnosis of asthma | GOLD guidelines recommend ICS-containing regimens for those with a prior diagnosis of asthma. This subgroup analysis seeks to examine the comparative effectiveness of Advair Diskus and HFA in this population where ICS-based regimens are thought to be particularly beneficial. | Addresses comparative effectiveness and safety in lower risk subgroup | None |
| **4** | Excluding patients with recent asthma diagnosis codes (in prior 3 years). | GOLD guidelines recommend ICS-containing regimens for those with a prior diagnosis of asthma. This subgroup analysis seeks to examine the comparative effectiveness of Advair Diskus and HFA in this population where ICS-based regimens are thought to be particularly beneficial. | Addresses comparative effectiveness and safety in lower risk subgroup | Low power |
| **6** | Spirometry before cohort entry | Maintenance inhalers are more likely to be effective for patients who have a proven diagnosis of COPD (as measured by spirometry). We want to examine whether, among patients with spirometry (i.e. patients most likely to benefit from inhaler therapy), there is any difference between Advair Diskus and HFA. | Addresses comparative effectiveness and safety in population enriched for COPD (closer to RCT population) | We do not know the actual measurements from the spirometry. |
| **7** | Index-prescription from a pulmonologist | Pulmonologists may be more likely to assign guideline-recommended therapy compared to other practitioners. We will examine whether Advair HFA has similar effectiveness to Advair Diskus among patients whose index prescription is from a pulmonologist. | Addresses generic quality in population enriched for patients who should be receiving guideline-concordant care. | Low power. |
| 8 | Cohort entry between 2007-2018 | This is the primary time during which Advair Diskus and Advair HFA were on the market without generic competition, limiting confounding from competing devices that patients might be alternatively prescribed. | Limits confounding from competing inhalers, providing a more direct comparison of Advair Diskus vs. HFA. | None. |
| 9 | Cohort entry between 2019-2023 | This study period includes both the approval of the first generic fluticasone-salmeterol (Wixela Inhub) in 2019, as well as the COVID-19 pandemic (starting in 2020). These events could potentially confound our estimate of the ATE due to changes in treatment selection, as well as respiratory outcomes, care, and hospitalizations due to COVID-19. | Allows us to observe how the ATE changes relative to our overall estimate during a period that includes COVID-19 and a major generic approval, which could flag potential confounding in the overall cohort. | Changes in ATE are likely due to unobserved confounders rather than comparative intrinsic effectiveness/safety between the Advair devices. |
| 10 | Gender (Male/Female) | COPD severity, incidence, and care has been observed to vary across various demographic dimensions; large observational studies such as this allow us to observe patients who are often excluded from clinical trials.^22–26^ | Allows us to observe potential heterogenous treatment effects between different demographic groups that often have inadequate representation in RCTs. | None. |
| 11 | Race (White/Black/  Hispanic/Asian) |  |  |  |
| 12 | Age (less than 65 vs. 65 and older) |  |  |  |

*Each subgroup analysis will be performed with patients who meet the definition (for example, at least 1 baseline COPD exacerbation) and those who do not meet the definition (for example, no baseline COPD exacerbations).

1. **Sensitivity analyses**

|  | **Sensitivity** | **Why?** | **Strengths** | **Weaknesses** |
| --- | --- | --- | --- | --- |
| **1** | Alter the grace period for continuous use (30 days, 90 days). | This allows us to assess whether estimates differ when the exposure definition changes. | Using a shorter grace period (30 days) will provide more specificity with respect to the exposure while the longer grace period (90 days) will better reflect real-world practice. | 30-day grace period will exclude people who are truly exposed, while 90-day grace period will include people are not truly exposed |
| **2** | ITT over one year of follow-up | We will assess whether there are differences between Advair Diskus and Advair HFA in a truly real-world setting in which patients frequently discontinue therapy. | This analysis will allow us to avoid informative censoring. | Much of the included person-time will be unexposed person-time. |
| **4** | Exclude the first 30 and 60 days after cohort entry. | Patients may receive ICS-LABAs when their symptoms are bad (but before a true moderate or severe COPD exacerbation). We will assess whether any differences between Advair Diskus and Advair HFA exist when we exclude these early exacerbations. | This allows us to focus on the long-term effectiveness of these maintenance therapies. | Follow-up time will be low because patients discontinue their inhalers so frequently. We may not have sufficient power to detect small treatment effects in this analysis. |
| **5** | Alter the definition of a moderate exacerbation: (a) require an office or ED visit before the prednisone fill; (b) require both prednisone (5-14 days) and a respiratory antibiotic; (c) count either a prednisone script (5-14 days) or a respiratory antibiotic script | This will allow us to assess the robustness of the results to variations of the outcome definition. | Each of these outcome definitions has been employed elsewhere in the literature. | (a) We will exclude many patients who have moderate COPD exacerbations but do not come into clinic. (b) We will exclude many patients who are having moderate exacerbations (antibiotics + steroids are only recommended for patients meeting specific criteria such as purulent sputum); (c) We will include patients who receive antibiotics for reasons having nothing to do with COPD exacerbations |
| **6** | Alter the definition of a severe exacerbation: allow for a COPD diagnosis in any position for the hospitalization | This will allow us to assess the robustness of the results to variations of the outcome definition. | The outcome definition in the primary effectiveness analysis may exclude patients who are having a COPD exacerbation but are admitted for a different primary reason (e.g. MI). | This is a less specific definition than the one we employed since many patients will have a code for COPD during a hospitalization even though they are not having an exacerbation. |
| **7** | Alter the definition of pneumonia hospitalization: require that the pneumonia diagnosis occur in the primary position for the hospitalization | This will allow us to assess the robustness of the results to variations of outcome definition. | The outcome definition in the primary safety analysis may capture patients with mild pneumonias. This will capture only the more severe pneumonias. | Pneumonias are a known side-effect of ICS-LABAs, and this sensitivity analysis will miss many clinically important pneumonias. |
| **8** | Logistic regression propensity score estimation | Given that we are using an augmented version of logistic regression to estimate propensity scores for our primary analysis, it will be informative to see how our estimates differ when we use standard logistic regression. | Allows comparison of our chosen propensity score estimation method with a standard method. | Might not be able to capture more complex relationships between the covariates (interactions, polynomial terms, etc.). |
| **9** | Entropy weighting | Balancing our cohort with a method that does not rely on propensity scores will allow us to gauge the robustness of our estimates to potential bias resulting from propensity score misspecification. | Balances covariates without the need to specify a propensity score, allowing us to gauge the robustness of our primary estimates. | Entropy weighted estimation can estimate a target estimand that deviates from the ATE in settings with high or low exposure prevalence.^27^ |
| **10** | High-dimensional propensity score matching | HdPS adjusts for hundreds of empirically defined covariates. | Rigorous, alternative methodology for controlling for confounding. | None |

**References**

1. Koser A, Westerman J, Sharma S, Emmett A, Crater GD. Safety and Efficacy of Fluticasone Propionate/Salmeterol Hydrofluoroalkane 134a Metered-Dose-Inhaler Compared with Fluticasone Propionate/Salmeterol Diskus in Patients with Chronic Obstructive Pulmonary Disease. *Open Respir Med J*. 2010;4:86-91. doi:10.2174/1874306401004010086

2. Gershon AS, Wang C, Guan J, Vasilevska-Ristovska J, Cicutto L, To T. Identifying Individuals with Physcian Diagnosed COPD in Health Administrative Databases. *COPD: Journal of Chronic Obstructive Pulmonary Disease*. 2009;6(5):388-394. doi:10.1080/15412550903140865

3. Rothnie KJ, Müllerová H, Hurst JR, et al. Validation of the Recording of Acute Exacerbations of COPD in UK Primary Care Electronic Healthcare Records. *PLOS ONE*. 2016;11(3):e0151357. doi:10.1371/journal.pone.0151357

4. Stein BD, Bautista A, Schumock GT, et al. The Validity of International Classification of Diseases, Ninth Revision, Clinical Modification Diagnosis Codes for Identifying Patients Hospitalized for COPD Exacerbations. *Chest*. 2012;141(1):87-93. doi:10.1378/chest.11-0024

5. Kern DM, Davis J, Williams SA, et al. Validation of an administrative claims-based diagnostic code for pneumonia in a US-based commercially insured COPD population. *International Journal of Chronic Obstructive Pulmonary Disease*. 2015;10:1417-1425. doi:10.2147/COPD.S83135

6. Imai K, Ratkovic M. Covariate Balancing Propensity Score. *Journal of the Royal Statistical Society Series B: Statistical Methodology*. 2014;76(1):243-263. doi:10.1111/rssb.12027

7. Greifer N. WeightIt: Weighting for Covariate Balance in Observational Studies. Published online 2024. Accessed February 22, 2024. https://github.com/ngreifer/WeightIt

8. Wyss R, Ellis AR, Brookhart MA, et al. The Role of Prediction Modeling in Propensity Score Estimation: An Evaluation of Logistic Regression, bCART, and the Covariate-Balancing Propensity Score. *American Journal of Epidemiology*. 2014;180(6):645-655. doi:10.1093/aje/kwu181

9. Austin PC, Stuart EA. The performance of inverse probability of treatment weighting and full matching on the propensity score in the presence of model misspecification when estimating the effect of treatment on survival outcomes. *Stat Methods Med Res*. 2017;26(4):1654-1670. doi:10.1177/0962280215584401

10. Austin PC. The performance of different propensity score methods for estimating marginal hazard ratios. *Stat Med*. 2013;32(16):2837-2849. doi:10.1002/sim.5705

11. Robins JM, Hernán MÁ, Brumback B. Marginal Structural Models and Causal Inference in Epidemiology: *Epidemiology*. 2000;11(5):550-560. doi:10.1097/00001648-200009000-00011

12. Austin PC. The use of propensity score methods with survival or time-to-event outcomes: reporting measures of effect similar to those used in randomized experiments. *Stat Med*. 2014;33(7):1242-1258. doi:10.1002/sim.5984

13. Austin PC, Stuart EA. Moving towards best practice when using inverse probability of treatment weighting (IPTW) using the propensity score to estimate causal treatment effects in observational studies. *Statistics in Medicine*. 2015;34(28):3661-3679. doi:10.1002/sim.6607

14. Ho D, Imai K, King G, Stuart EA. MatchIt: Nonparametric Preprocessing for Parametric Causal Inference. *Journal of Statistical Software*. 2011;42:1-28. doi:10.18637/jss.v042.i08

15. Whittaker H, Rubino A, Müllerová H, et al. Frequency and Severity of Exacerbations of COPD Associated with Future Risk of Exacerbations and Mortality: A UK Routine Health Care Data Study. *Int J Chron Obstruct Pulmon Dis*. 2022;17:427-437. doi:10.2147/COPD.S346591

16. Yang W, Jepson C, Xie D, et al. Statistical Methods for Recurrent Event Analysis in Cohort Studies of CKD. *Clin J Am Soc Nephrol*. 2017;12(12):2066-2073. doi:10.2215/CJN.12841216

17. Sagara I, Giorgi R, Doumbo OK, Piarroux R, Gaudart J. Modelling recurrent events: comparison of statistical models with continuous and discontinuous risk intervals on recurrent malaria episodes data. *Malar J*. 2014;13:293. doi:10.1186/1475-2875-13-293

18. Amorim LD, Cai J. Modelling recurrent events: a tutorial for analysis in epidemiology. *Int J Epidemiol*. 2015;44(1):324-333. doi:10.1093/ije/dyu222

19. Prentice RL, Williams BJ, Peterson AV. On the regression analysis of multivariate failure time data. *Biometrika*. 1981;68(2):373-379. doi:10.1093/biomet/68.2.373

20. Cole SR, Hernán MA. Adjusted survival curves with inverse probability weights. *Computer Methods and Programs in Biomedicine*. 2004;75(1):45-49. doi:10.1016/j.cmpb.2003.10.004

21. Xie J, Liu C. Adjusted Kaplan–Meier estimator and log‐rank test with inverse probability of treatment weighting for survival data. *Statistics in Medicine*. 2005;24(20):3089-3110. Accessed February 19, 2024. https://onlinelibrary.wiley.com/doi/10.1002/sim.2174

22. Cecere LM, Slatore CG, Uman JE, et al. Adherence to Long-Acting Inhaled Therapies among Patients with Chronic Obstructive Pulmonary Disease (COPD). *COPD: Journal of Chronic Obstructive Pulmonary Disease*. 2012;9(3):251-258. doi:10.3109/15412555.2011.650241

23. Eisner MD, Blanc PD, Omachi TA, et al. Socioeconomic status, race and COPD health outcomes. *Journal of Epidemiology & Community Health*. 2011;65(1):26-34. doi:10.1136/jech.2009.089722

24. Monteiro C, Maricoto T, Prazeres F, Augusto Simões P, Augusto Simões J. Determining factors associated with inhaled therapy adherence on asthma and COPD: A systematic review and meta-analysis of the global literature. *Respiratory Medicine*. 2022;191:106724. doi:10.1016/j.rmed.2021.106724

25. Mamary AJ, Stewart JI, Kinney GL, et al. Race and Gender Disparities are Evident in COPD Underdiagnoses Across all Severities of Measured Airflow Obstruction. *Chronic Obstr Pulm Dis*. 5(3):177-184. doi:10.15326/jcopdf.5.3.2017.0145

26. Matera MG, Ora J, Calzetta L, Rogliani P, Cazzola M. Sex differences in COPD management. *Expert Review of Clinical Pharmacology*. 2021;14(3):323-332. doi:10.1080/17512433.2021.1888713

27. Austin PC. Differences in target estimands between different propensity score-based weights. *Pharmacoepidemiology and Drug Safety*. 2023;32(10):1103-1112. doi:10.1002/pds.5639
