# Supplementary material for: The comparative effectiveness and safety of fluticasone-salmeterol via metered-dose versus dry powder inhalers for COPD: A new user cohort study
Source: PLoS Med. 2025 May 14;22(5):e1004596. doi: 10.1371/journal.pmed.1004596 (PMC12077913; doi:10.1371/journal.pmed.1004596)
Supplement: S1 Methods — (DOCX) [file pmed.1004596.s003.docx]

**S1 Methods.**

Covariates

We measured covariates in the 180 days prior to and including the date of cohort entry, unless otherwise specified. All of the following covariates were included in our propensity score model: (1) COPD severity, including the number of baseline moderate COPD exacerbations (requiring a prednisone prescription but no hospitalization), number of baseline severe COPD exacerbations (requiring hospitalization), number of pneumonia hospitalizations, prior asthma diagnosis (using all available data), respiratory antibiotics, maintenance inhaler therapy (long-acting muscarinic antagonists [LAMA], long-acting β-agonists [LABA], inhaled corticosteroids [ICS], LAMA-LABAs, ICS-LAMA-LABAs), rescue inhaler therapy (short-acting β-agonists [SABAs] and short-acting muscarinic antagonists [SAMAs]), long-term oral therapy for COPD (azithromycin, roflumilast), continuous or bilevel positive airway pressure, use of spirometry, smoking, use of home oxygen therapy, and pulmonary rehabilitation; (2) other co-morbidities, including a combined comorbidity score [55], a frailty index score [56], obstructive sleep apnea, hypertension, diabetes, obesity, coronary artery disease, peripheral vascular disease, venous thromboembolic disease, congestive heart failure, gastroesophageal reflux disease, renal failure, osteoporosis, neurologic disease, cancer (non-metastatic and metastatic), anxiety disorders, and depression; (3) baseline healthcare utilization, including the number of emergency department visits, hospitalizations, 90-day readmissions, office visits, pulmonology office visits, prescription drug claims, basic metabolic or complete metabolic panels, complete blood counts, electrocardiograms, echocardiograms, computed tomography scans, bronchoscopies, colonoscopies, mammography, bone mineral density scans, and influenza vaccination; (4) use of non-pulmonary medications, including statins, β-blockers, angiotensin-converting enzyme inhibitors, angiotensin-receptor blockers, calcium-channel blockers, thiazide diuretics, loop diuretics, proton-pump inhibitors, H_2_-receptor blockers, metformin, sulfonylureas, sodium–glucose co-transporter-2 [SGLT2] inhibitors, dipeptidyl peptidase-4 [DPP-4] inhibitors, glucagon-like peptide 1 [GLP-1] agonists, benzodiazepines, and selective serotonin reuptake inhibitors or serotonin–norepinephrine reuptake inhibitors.

We assessed demographic characteristics (age, sex, race, and region), year of cohort entry, and whether index prescriptions were written by a pulmonologist on the index date. We also assessed whether patients had moderate or severe COPD exacerbations and whether patients filled prescriptions for respiratory antibiotics and/or prednisone in the 30 days leading up to and including cohort entry.

Propensity Score Model Selection

Based on the diagnostic metrics collected during our design phase and presented in S2 Table, we selected an over-identified version of the covariate balancing propensity score (CBPS) given its strong performance in balancing covariates in our dataset while displaying a slightly lesser degree of weight inflation than other propensity score models. This model augments standard logistic regression propensity score estimation by replacing the maximum likelihood estimator with a generalized method of moments estimator that imposes additional balance constraints, iteratively “nudging” coefficient estimates toward a model that maximizes covariate balance between groups [17,22]. CBPS still shares the same limitations of logistic regression propensity scores in that its performance is limited by the degree to which all relevant covariates are included and the functional form of the model is correctly specified. However, past work has demonstrated that CBPS is generally more robust in balancing covariates and reducing bias relative to mis-specified logistic propensity score models while still maintaining greater ease of interpretability and relatively lower computational intensity compared to gradient boosted models [22].

Outcome Models

We estimated HRs and 95% CIs for time-to-event outcomes (including the primary outcome measures, time to first moderate exacerbation, time to first severe exacerbation, and time to death) using a Cox proportional hazards model regressing the outcome on exposure as the sole predictor and adjusted using weights derived from CBPS-IPTW. Separate covariate terms were not included in the outcome models given that any confounding from those covariates is controlled for through the IPTW adjustment. The same is true for our negative binomial model used to assess annual rates of moderate or severe COPD exacerbations and annual rates of pneumonia hospitalizations as secondary endpoints. We checked the proportionality assumption for our Cox models using Schoenfeld residual score tests and log-minus-log (LML) plots [33].
