# Supplementary material for: The comparative effectiveness and safety of fluticasone-salmeterol via metered-dose versus dry powder inhalers for COPD: A new user cohort study
Source: PLoS Med. 2025 May 14;22(5):e1004596. doi: 10.1371/journal.pmed.1004596 (PMC12077913; doi:10.1371/journal.pmed.1004596)
Supplement: S1 Table — a. For every covariate Xi, the distributions of their quadratic and cubic transformations (i.e., Xi2 and Xi3) as well as their two-way interactions with other covariates (i.e., Xi*Xj) were compared between the exposure and referent groups using standardized mean differences, and the maximum standardized mean difference (i.e., imbalance) among these higher-order terms is reported for each propensity score model. The aim was to balance on higher-order covariate moments and interactions to help achieve optimal balance in observational studies [20,21]. CBPS: covariate-balancing propensity score; GBM: generalized boosted model; GLM: generalized linear model (logistic regression). (DOCX) [file pmed.1004596.s004.docx]

**S1 Table. Target trial emulation protocol.**

| **Protocol Element** | **Description** | **Target Trial** | **Emulation with Observational Data from** **Optum’s Clinformatics DataMart** |
| --- | --- | --- | --- |
| Eligibility criteria | Who will be included in this study? | Patients aged 40 years or older with COPD newly initiating fluticasone-salmeterol metered-dose inhalers (Advair HFA) or fluticasone-salmeterol (Advair Diskus). | Same as target trial |
| Treatment strategies | Which precise treatment strategies or interventions will eligible individuals receive? | 1. Initiate fluticasone-salmeterol metered-dose inhaler (Advair HFA)  2. Initiate fluticasone-salmeterol dry powder inhaler (Advair Diskus) | Same as target trial |
| Treatment assignment | How will eligible individuals be assigned to the treatment strategies? | Randomization, no blinding | Eligible individuals assigned at baseline to the treatment strategy consistent with their prescription. To emulate randomization, we adjust for a large set of relevant confounders using IPTW with a CBPS model. Confounders include COPD severity, comorbidities, healthcare utilization, and medications (see S1 Methods). |
| Outcomes | What outcomes will be measured during follow-up? | 1. Time to first moderate or severe COPD exacerbation  2. Time to first hospitalization for pneumonia  3. Secondary effectiveness and safety outcomes, including:   - Time to first moderate exacerbation - Time to first severe exacerbation - Annual rate of moderate or severe COPD exacerbations - Annual rate of moderate exacerbation - Annual rate of severe exacerbations - Annual rate of pneumonia hospitalizations - Time to all-cause mortality | Same as target trial. Outcomes identified through claims data (ICD-9/10 codes, prescriptions). Definitions for moderate/severe exacerbations and pneumonia hospitalizations align with target trial specifications. |
| Causal estimand | Which causal estimand will be estimated with the observational data? | Per protocol average treatment effect (effect of receiving treatment strategy as specified in protocol). | Per protocol, on-treatment average treatment effect (effect of receiving treatment strategy as specified in protocol) |
| Start and end of follow-up | When does follow-up start and when does it end? | Starts at randomization and ends at 1 year, discontinuation of therapy, switch of therapy, death, end of insurance coverage, or administrative censoring. | Starts at index prescription initiation and ends at 1 year, discontinuation of therapy (with a 60-day grace period), therapy switch, death, or administrative censoring. |
| Statistical analysis | Which statistical analyses will be used to estimate the causal estimand? | Per protocol analysis using hazard ratios from Cox proportional hazards models and rates from negative binomial models, adjusting for baseline covariates via IPTW. | Same as target trial. Stabilized IPTW based on CBPS model is used to balance covariates. Sensitivity analyses and alternative outcome definitions are implemented to check robustness.^a^ |

LAMA: long-acting muscarinic antagonist; ICS: inhaled corticosteroid; LABA: long-acting beta agonist; CBPS: covariate-balancing propensity score; IPTW: inverse probability of treatment weighting.

This table outlines the design elements of a target trial emulation, comparing the idealized design of a randomized controlled trial (target trial) to the corresponding observational emulation conducted using real-world data. The purpose is to align observational study methods with the principles of causal inference in a transparent fashion, highlighting key assumptions, design features, and analytical approaches to estimate causal effects [23].

a. A more detailed description of the statistical analysis can be found in the corresponding paper.
