# Supplementary material for: The comparative effectiveness and safety of fluticasone-salmeterol via metered-dose versus dry powder inhalers for COPD: A new user cohort study
Source: PLoS Med. 2025 May 14;22(5):e1004596. doi: 10.1371/journal.pmed.1004596 (PMC12077913; doi:10.1371/journal.pmed.1004596)
Supplement: S2 Table — This table outlines the design elements of a target trial emulation, comparing the idealized design of a randomized controlled trial (target trial) to the corresponding observational emulation conducted using real-world data. The purpose is to align observational study methods with the principles of causal inference in a transparent fashion, highlighting key assumptions, design features, and analytical approaches to estimate causal effects [23]. a. A more detailed description of the statistical analysis can be found in the corresponding paper. LAMA: long-acting muscarinic antagonist; ICS: inhaled corticosteroid; LABA: long-acting beta agonist; CBPS: covariate-balancing propensity score; IPTW: inverse probability of treatment weighting. (DOCX) [file pmed.1004596.s005.docx]

**S2 Table. Relative performance of inverse probability of treatment weighting between selected propensity score models in balancing covariates.**

| **Propensity Score Model** | **Effective Sample Size of Exposure Group** | **Mean standardized mean difference** | **Maximum standardized mean difference** | **Maximum standardized mean difference across 2^nd^ or 3^rd^ degree polynomial terms and interaction terms^a^** | **Ratio between highest and lowest weight (minimum to maximum weight)** | **Mean weight (interquartile range)** |
| --- | --- | --- | --- | --- | --- | --- |
| CBPS | 13,326 | 0.0073 | 0.0503 | 0.0710 | 61  (0.14-8.31) | 1.00  (0.92-0.99) |
| GBM | 13,061 | 0.0074 | 0.0332 | 0.0578 | 65  (1.00-70.54) | 1.94  (1.06-1.14) |
| GLM | 12,896 | 0.0089 | 0.0742 | 0.0748 | 69  (1.01-78.58) | 2.00  (1.05-1.14) |
| Unadjusted | 24,060 | 0.0785 | 0.5380 | 0.5685 | NA | NA |

CBPS: covariate-balancing propensity score; GBM: generalized boosted model; GLM: generalized linear model (logistic regression).

a. For every covariate X_i_, the distributions of their quadratic and cubic transformations (i.e., X_i_^2^ and X_i_^3^) as well as their two-way interactions with other covariates (i.e., X_i_*X_j_) were compared between the exposure and referent groups using standardized mean differences, and the maximum standardized mean difference (i.e., imbalance) among these higher-order terms is reported for each propensity score model. The aim was to balance on higher-order covariate moments and interactions to help achieve optimal balance in observational studies [20,21].
