# Supplementary material for: The comparative effectiveness and safety of fluticasone-salmeterol via metered-dose versus dry powder inhalers for COPD: A new user cohort study
Source: PLoS Med. 2025 May 14;22(5):e1004596. doi: 10.1371/journal.pmed.1004596 (PMC12077913; doi:10.1371/journal.pmed.1004596)
Supplement: S3 Table — a. Median follow-up time in the group of patients receiving Advair Diskus was 88 days (interquartile range [IQR] 88–156 days). Mean follow-up time was 132 days (standard deviation [SD] 96 days). b. Median follow-up time in the group of patients receiving Advair HFA was 88 days (IQR 72–148 days). Mean follow-up time was 120 days (SD 90 days). LAMA: long-acting muscarinic antagonist; ICS: inhaled corticosteroid; LABA: long-acting beta agonist. (DOCX) [file pmed.1004596.s006.docx]

**S3 Table. Reasons for censoring in the analysis of first moderate or severe COPD exacerbation.**

| **Censoring reason** | **Advair Diskus (n=177,992), n (%)^a^** | **Advair HFA (n=24,060), n (%)^b^** |
| --- | --- | --- |
| Outcome | 27,031 (15.2) | 3,550 (14.8) |
| Death | 5,067 (2.8) | 691 (2.9) |
| End of patient enrollment | 13,329 (7.5) | 1,547 (6.4) |
| Start of exposure different from the index exposure | 728 (0.4) | 421 (1.7) |
| LAMA, LAMA, ICS, ICS-LABA, or LAMA-LABA begun | 14,110 (7.9) | 2,778 (11.5) |
| End of index exposure | 103,760 (58.3) | 12,956 (53.8) |
| Maximum follow-up time | 13,417 (7.5) | 1,314 (5.5) |
| End of data | 550 (0.3) | 803 (3.3) |

LAMA: long-acting muscarinic antagonist; ICS: inhaled corticosteroid; LABA: long-acting beta agonist.

a. Median follow-up time in the group of patients receiving Advair Diskus was 88 days (interquartile range [IQR] 88-156 days). Mean follow-up time was 132.1 days (standard deviation [SD] 96.1 days).

b. Median follow-up time in the group of patients receiving Advair HFA was 88 days (IQR 72-148 days). Mean follow-up time was 119.9 days (SD 90.0 days).
