# Supplementary material for: The comparative effectiveness and safety of fluticasone-salmeterol via metered-dose versus dry powder inhalers for COPD: A new user cohort study
Source: PLoS Med. 2025 May 14;22(5):e1004596. doi: 10.1371/journal.pmed.1004596 (PMC12077913; doi:10.1371/journal.pmed.1004596)
Supplement: S4 Table — a. Median follow-up time in the group of patients receiving Advair Diskus was 88 days (interquartile range [IQR] 88–177 days). Mean follow-up time was 144 days (standard deviation [SD] 100 days). b. Median follow-up time in the group of patients receiving Advair HFA was 88 days (IQR 88–155 days). Mean follow-up time was 131 days (SD 95 days). LABA: long-acting beta agonist; LAMA: long-acting muscarinic antagonist; ICS: inhaled corticosteroid. (DOCX) [file pmed.1004596.s007.docx]

**S4 Table. Reasons for censoring in the analysis of first pneumonia hospitalization.**

| **Censoring reason** | **Advair Diskus (n=177,992), n (%)^a^** | **Advair HFA (n=24,060), n (%)^b^** |
| --- | --- | --- |
| Outcome | 8,239 (4.6) | 1,089 (4.5) |
| Death | 4,799 (2.7) | 612 (2.5) |
| End of patient enrollment | 14,530 (8.2) | 1,673 (7.0) |
| Start of exposure different from the index exposure | 823 (0.5) | 489 (2.0) |
| LABA, LAMA, ICS, ICS-LABA, or LAMA-LABA begun | 16,357 (9.2) | 3,170 (13.2) |
| End of index exposure | 115,191 (64.7) | 14,434 (60.0) |
| Maximum follow-up time | 17,439 (9.8) | 1,717 (7.1) |
| End of data | 614 (0.3) | 876 (3.6) |

LABA: long-acting beta agonist; LAMA: long-acting muscarinic antagonist; ICS: inhaled corticosteroid.

a. Median follow-up time in the group of patients receiving Advair Diskus was 88 days (interquartile range [IQR] 88-177 days). Mean follow-up time was 144.2 days (standard deviation [SD] 100.2 days).

b. Median follow-up time in the group of patients receiving Advair HFA was 88 days (IQR 88-155 days). Mean follow-up time was 130.9 days (SD 94.7 days).
