# Supplementary figures and images for: The comparative effectiveness and safety of fluticasone-salmeterol via metered-dose versus dry powder inhalers for COPD: A new user cohort study
Source: PLoS Med. 2025 May 14;22(5):e1004596. doi: 10.1371/journal.pmed.1004596 (PMC12077913; doi:10.1371/journal.pmed.1004596)

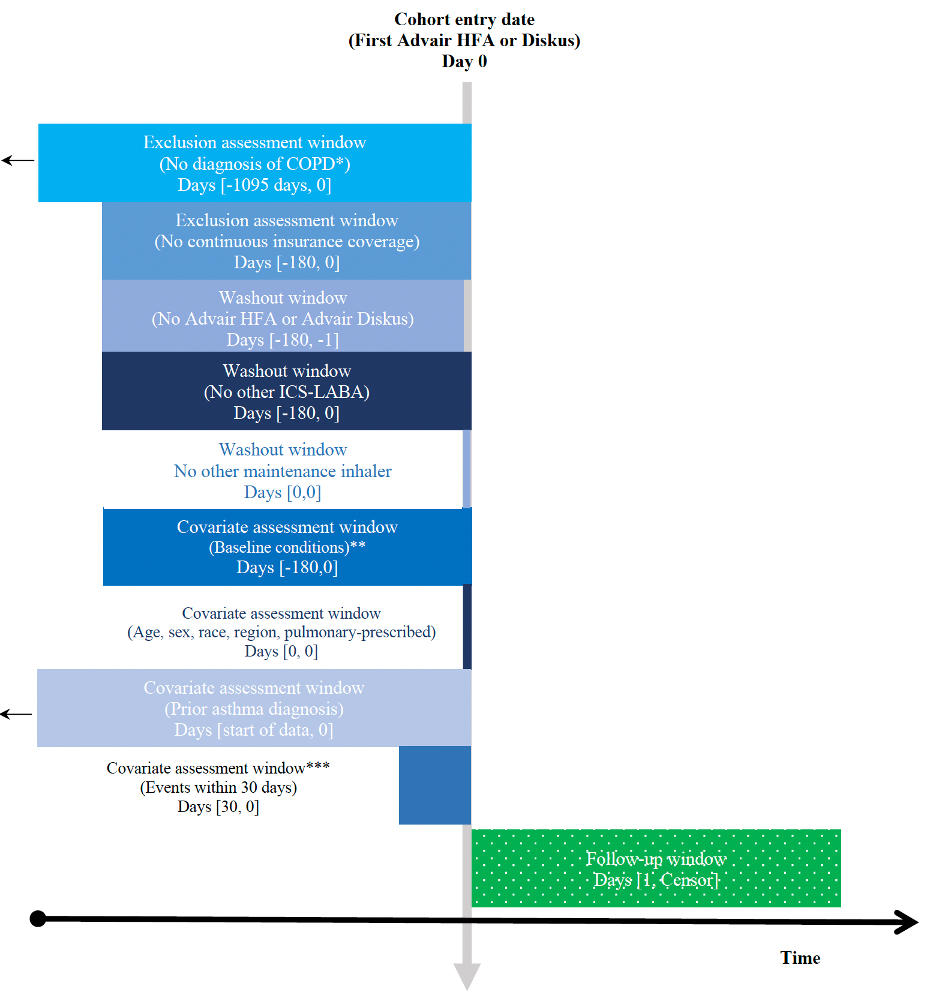

Supplement: S1 Fig — This graphical representation of study design shows how exclusion criteria were applied prior to cohort entry and how covariates were assessed [1]. *COPD diagnoses were based on 1 inpatient claim or 3 outpatient claims in the 3 years before cohort entry. **This includes all covariates except events within 30 days, asthma diagnosis codes, and smoking diagnosis codes. ***Events within 30 days include COPD exacerbations, fills of respiratory antibiotics, and fills of prednisone. ICS: inhaled corticosteroid; LABA: long-acting beta-2 agonist. (TIFF) [file pmed.1004596.s008.tiff]

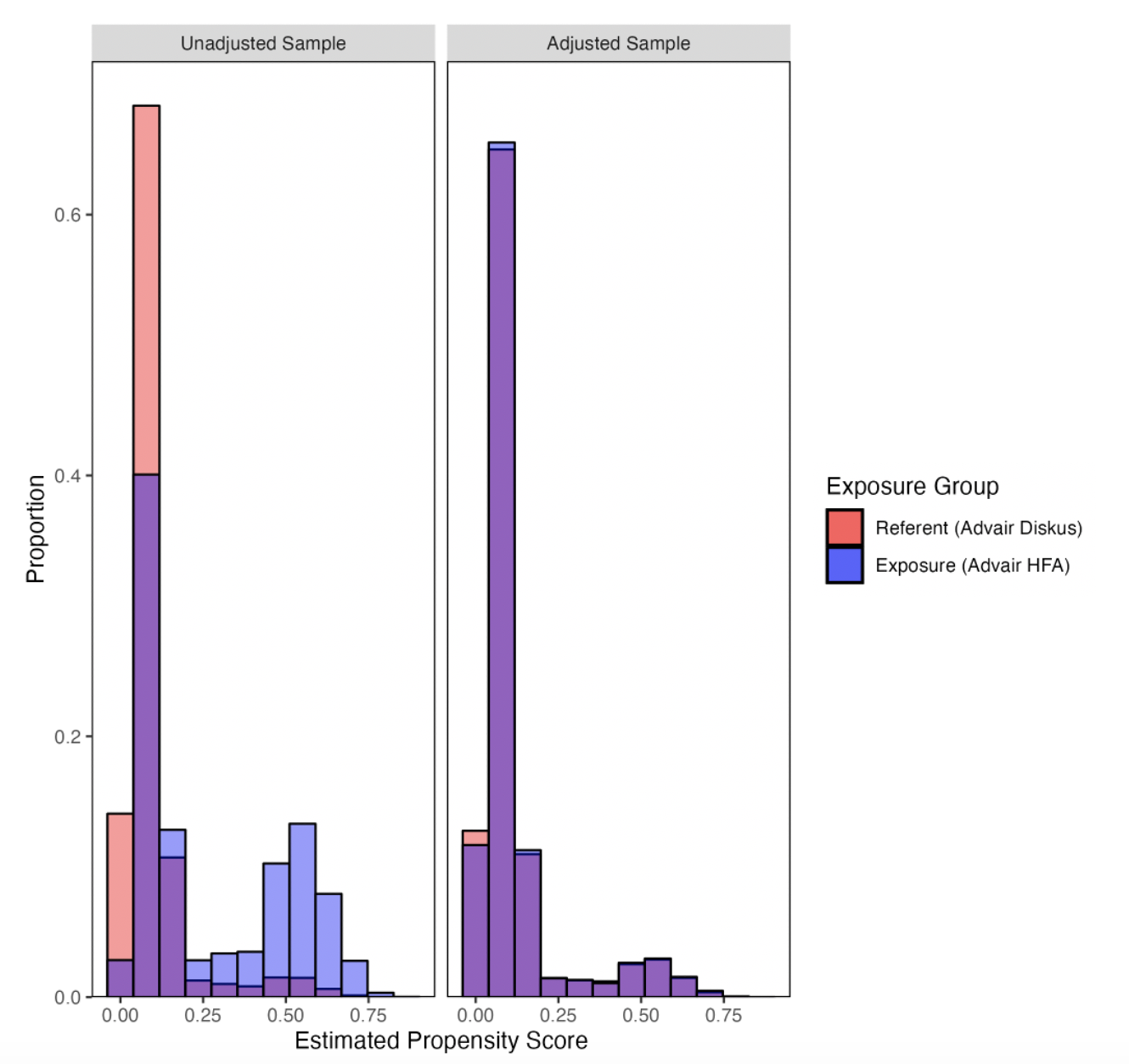

Supplement: S2 Fig — These figures show propensity score distributions between the exposure (Advair HFA) and referent (Advair Diskus) groups before and after adjustment with inverse probability weighting using a covariate-balancing propensity score (CBPS) model. The blue plot captures the propensity score distribution of new users of Advair HFA, the red plot captures the propensity score distribution of new users of Advair Diskus, and purple indicates overlap between the two distributions. The pre-weighting c-statistic was calculated to be 0.766 and the post-weighting c-statistic was calculated to be 0.527. (TIFF) [file pmed.1004596.s009.tiff]

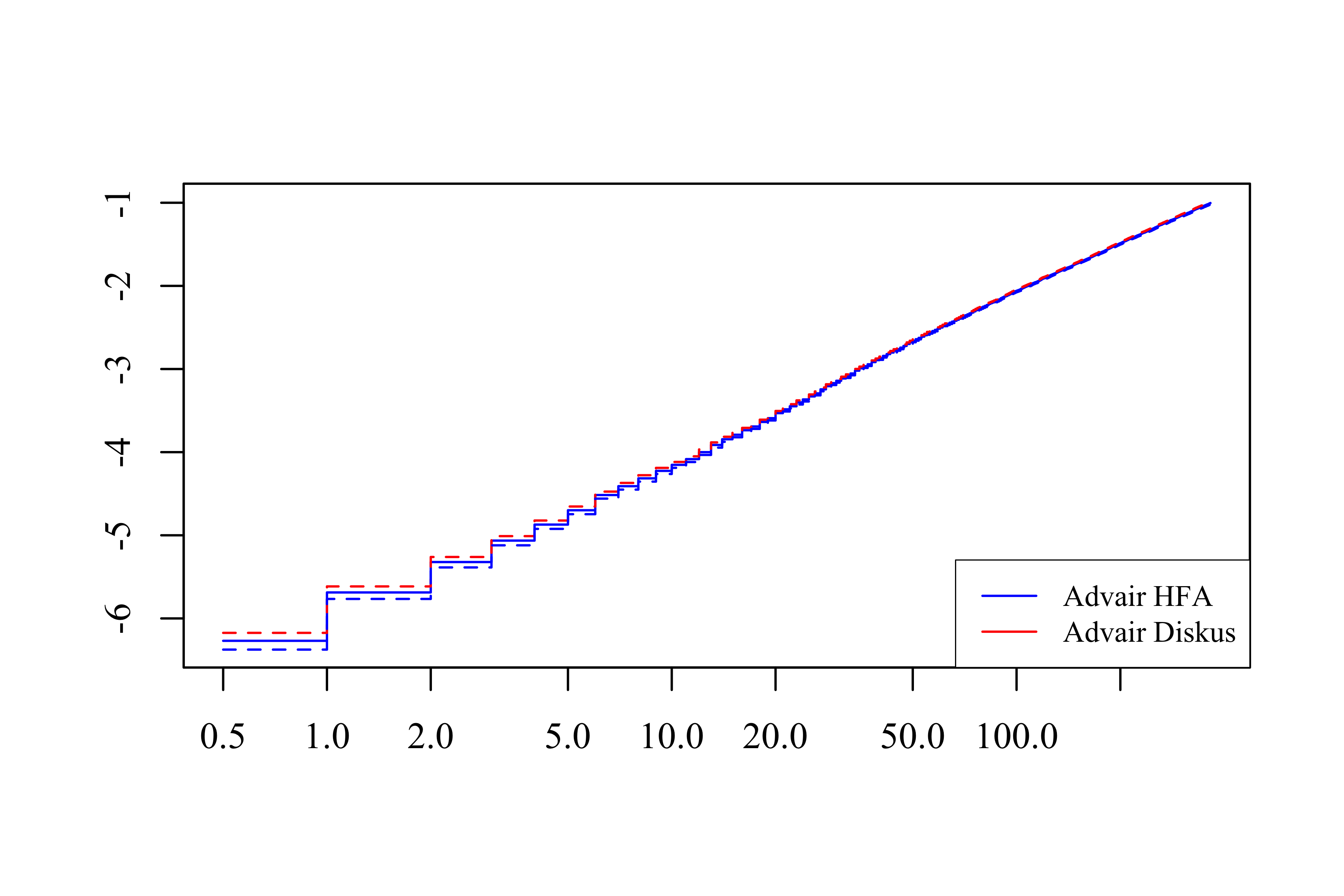

Supplement: S3 Fig — This figure shows the log-minus-log plot of the Cox proportional hazards model for first moderate or severe COPD exacerbation in new users of fluticasone-salmeterol metered-dose (Advair HFA) versus dry powder (Advair Diskus) inhalers. Parallel curves imply a constant hazard ratio and provides evidence that supports the proportional hazard assumption of the Cox model [33]. (TIFF) [file pmed.1004596.s010.tiff]

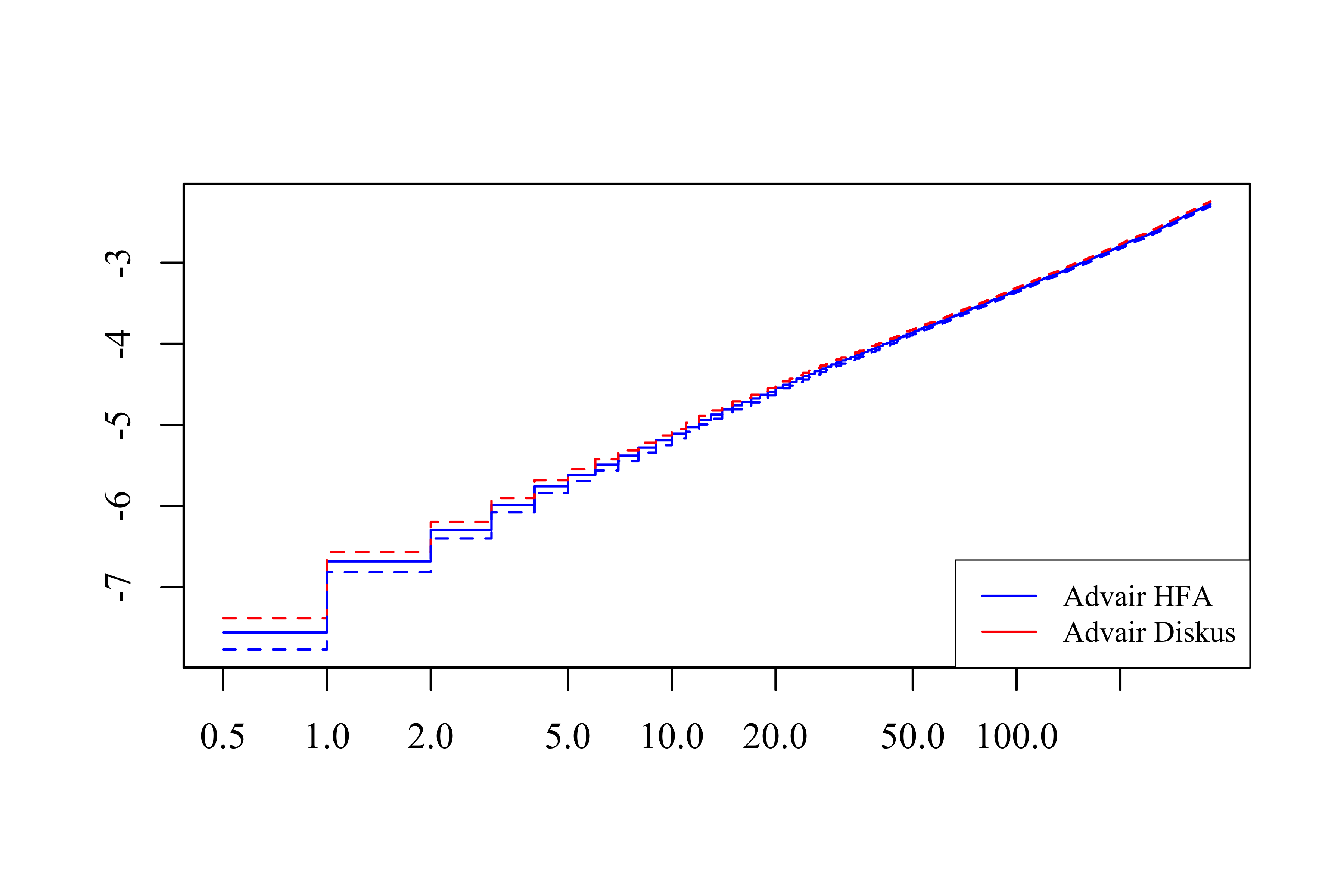

Supplement: S4 Fig — This figure shows the log-minus-log plot of the Cox proportional hazards model for first pneumonia hospitalization in new users of fluticasone-salmeterol metered-dose (Advair HFA) versus dry powder (Advair Diskus) inhalers. Parallel curves imply a constant hazard ratio and provides evidence that supports the proportional hazard assumption of the Cox model [33]. (TIFF) [file pmed.1004596.s011.tiff]

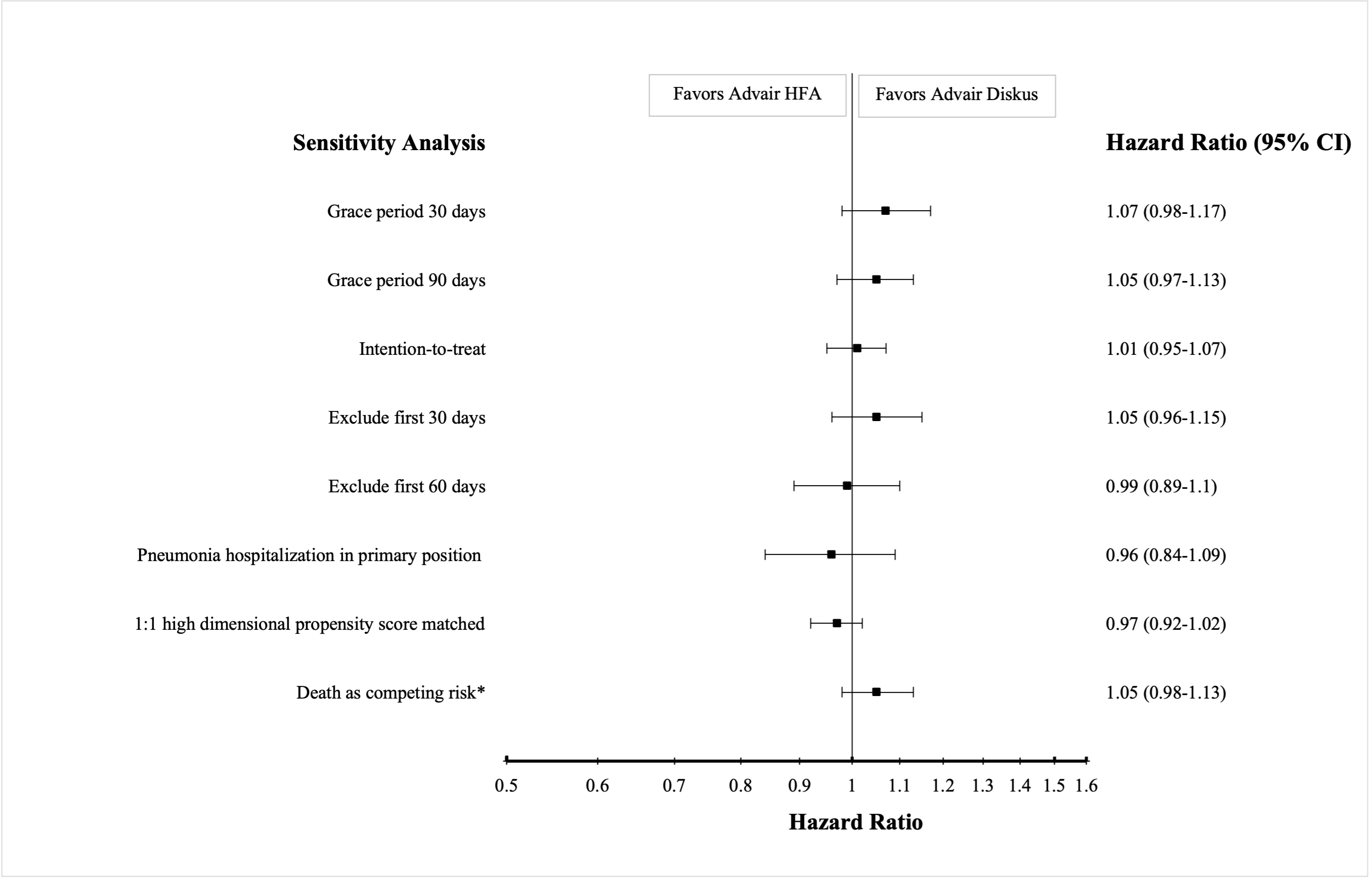

Supplement: S5 Fig — This figure shows the hazard ratios and 95% confidence intervals of first pneumonia hospitalization in new users of fluticasone-salmeterol metered-dose (Advair HFA) versus dry powder (Advair Diskus) inhalers across a range of prespecified sensitivity analyses. Hazard ratios greater than 1 mean that patients receiving fluticasone-salmeterol metered-dose inhalers have a higher hazard of first pneumonia hospitalization. *The output of the Cox-based model with death as a competing risk is a risk ratio rather than a hazard ratio, with bootstrap-generated confidence intervals. (TIFF) [file pmed.1004596.s012.tiff]

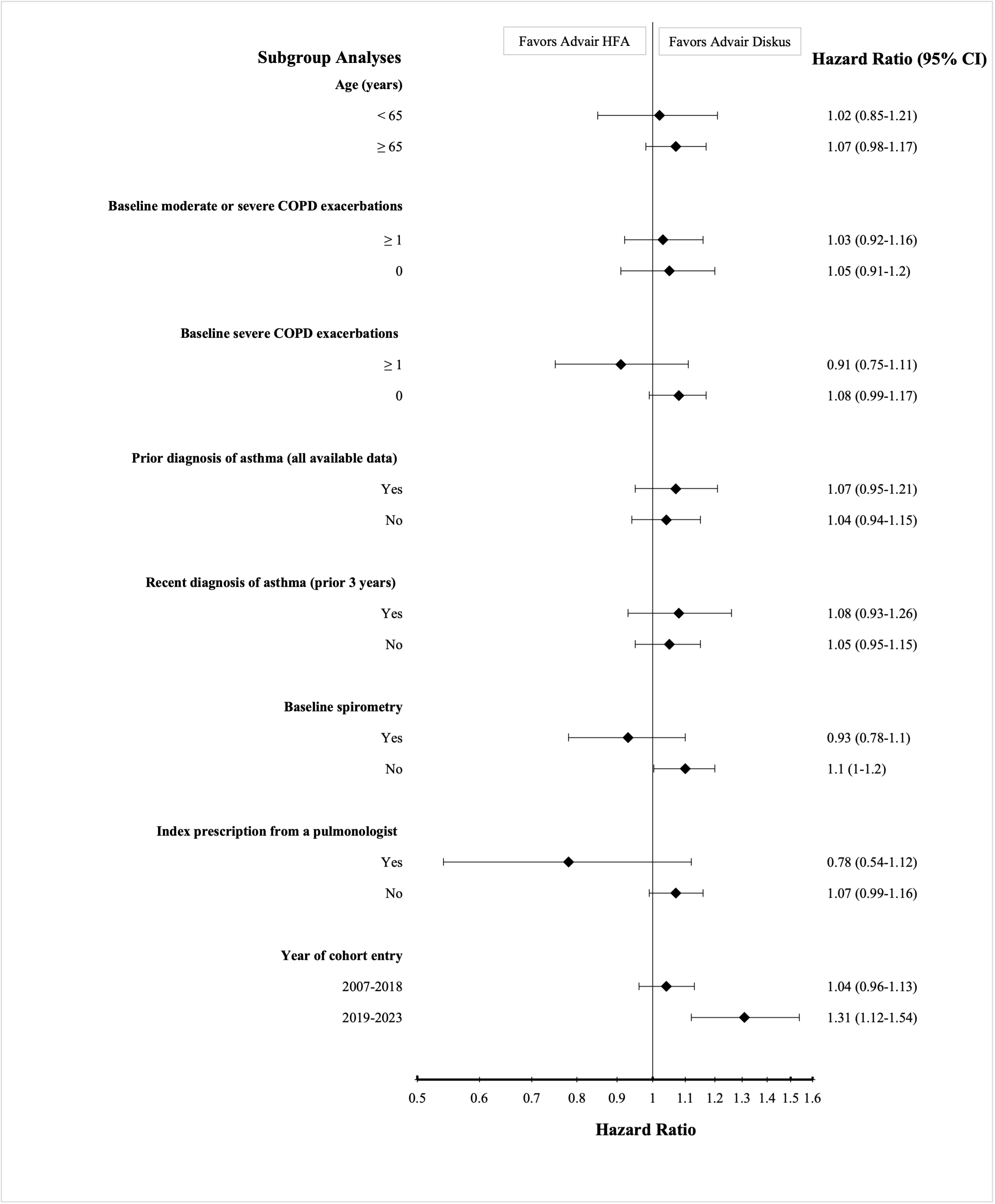

Supplement: S6 Fig — This figure shows the hazard ratios and 95% confidence intervals of first pneumonia hospitalization in new users of fluticasone-salmeterol metered-dose (Advair HFA) versus dry powder (Advair Diskus) inhalers across a range of prespecified subgroup analyses. Hazard ratios greater than 1 mean that patients receiving fluticasone-salmeterol metered-dose inhalers have a higher hazard of first pneumonia hospitalization. (TIFF) [file pmed.1004596.s013.tiff]
